# Distinct roles of dopamine receptors in HIV latency reversal in a myeloid cell model

**DOI:** 10.3389/fimmu.2026.1817754

**Published:** 2026-05-28

**Authors:** Liana V. Basova, Tera Riley, Violaine Delorme-Walker, Daniel de Siqueira-Lima, Richard Milner, Ronald J. Ellis, Howard Fox, Maria Cecilia Garibaldi Marcondes

**Affiliations:** 1 San Diego Biomedical Research Institute, San Diego, CA, United States; 2 National Institute for Drug Abuse, Summer Internship, 2023, San Diego, CA, United States; 3 Department of Psychiatry, University of California, San Diego, San Diego, CA, United States; 4 Department of Neurological Sciences, University of Nebraska Medical Center, Omaha, NE, United States

**Keywords:** Dopamine, dopamine receptors, HIV, latency, latency reversal, substance use

## Abstract

**Background:**

Currently, studies on the interactions between neurotransmitters and immune cells that are targets of HIV are limited by a focus on single pathways at a time. The latent myeloid reservoir cells in the brain are a source of virus responsive to changes in the levels of dopamine (DA), caused by stimulant drugs such as methamphetamine (Meth), which, in part, explains the poor control of brain viral load in models of chronic Meth exposure. Among the effects of DA, HIV latency reversal is observed in a proportion of latent cells that turn on the transcription of viral genes. In this study, our goal was to define the specific DA receptors and pathways involved in latency reversal by DA, under the hypothesis that downstream effects of DA activating HIV transcription can be learned and serve as targets for future therapeutic purposes.

**Methods:**

We focused on DA receptor (DRD)1 and DRD4, using the selective agonists SKF38393 hydrobromide and PD168077 maleate, which, like DA, increased levels of p24 in culture supernatants. A single-cell approach identified cells and enriched phenotypes associated with positive transcription of HIV genes.

**Results:**

Interestingly, we found very little overlap between DA and the individual receptor clusters, pathways, and upstream regulators, while the two receptors exhibited higher phenotypic cluster similarities and a higher number of perturbed genes indicating cross-regulation. Yet, between conditions, different pathways were convergent in isolated signatures and by their overall involvement in inflammation.

**Discussion:**

While Meth was a major contextual motivation for this work, the implications of these findings extend beyond stimulant use disorders, indicating that neuroimmune interactions are complex with resulting phenotypes derived from a combination of signaling pathways. The results suggest that latency reversal by DA in myeloid targets, especially in the brain, can occur with the contribution of DRDs’ signaling via a diversity of inflammatory pathways that have cell activation as a common feature.

## 1 Introduction

We have previously shown that dopamine (DA), a neurotransmitter involved in reward and addiction, and strongly elevated by stimulant substance use, acts on myeloid immune cells modifying phenotypes and responses to viral infections such as human immunodeficiency virus (HIV) (1, 2). Innate immune cells, such as microglia and macrophages, are major targets of HIV infection in the central nervous system (CNS), where the virus evolves to a latent state, making the brain a critical viral reservoir (3–6). Importantly, these HIV target cells express DA receptors (DRDs), making them highly susceptible to changes in the neurotransmitter environment, including by increasing the co-receptor for viral entry CCR5 and enhancing viral transcription in latent cells (7–11). This is relevant to persons living with HIV (PWH) who are users of stimulant substances such as methamphetamine (Meth). In addition to the challenges in treating this population due to their poor adherence to suppressive antiretrovirals (ARVs) (12), the high levels of DA in the brain affect HIV-infected reservoir cells, potentially contributing to higher viral load in the brain (13, 14), to chronic neuroinflammation (11, 15), and to the aggravated neurocognitive impairment (NCI) in this population (16–19).

In the brain, latent reservoir cells are a source of virus that can expand and repopulate other sites (4, 20, 21). Our previous findings have indicated that the stimulation of monocytes via different DA receptors can reverse latency, with a particularly interesting contribution of type I DA receptors such as (DRD)1 and a type II DRD4 (7), which are expressed by HIV myeloid target cells. This finding has implications to controlling HIV chronic infection and neuroinflammation in the context of substance use. Effects of DA enhancing HIV infection were attributed to the ability of DRD1 to decrease CCR5 expression by acting epigenetically on its promoter, while DRD4 increased its availability (7). Most importantly, DA also influenced HIV latency in a promonocyte cell line system, activating the transcription of viral genes in a significant subpopulation of cells (1). Calprotectin (MRP8/14) emerged as a DA-induced mediator of latency reversal and a biomarker that can identify Meth users with uncontrolled viral load (2), with implications for prognosis and detection and for control of the virus in the challenging population of Meth users. However, the mechanisms contributing to the effects of DA on latency in myeloid targets are not known.

In this study, we aimed at understanding the underlying causes of DA-induced latency reversal via DRD1 and DRD4 while generating transcriptional profiles compatible with pathogenesis. For this, we used a stable U1 promonocyte latency model, where all cells contain integrated HIV sequences (22), and stimulation with selective receptor agonists—SKF38393 hydrobromide and PD168077 maleate, respectively—compared to vehicle controls, and in relation to DA. A single-cell transcriptomics approach allowed the computational sorting of cells induced by these stimuli to transcribe HIV genes, for the identification of exclusive and overlapping signatures. The results indicated that despite the fact that both receptors mimicked DA in inducing latency reversal, very little signature overlap occurred, suggesting that a diversity of inflammatory pathways can induce HIV gene transcription in latent cells triggered via DRDs.

## 2 Material and methods

### 2.1 Promonocytic cell lines, culture conditions and p24 levels

Chronically infected HIV-1 (23) promonocytic cell lines (U1 and ARP-165) were obtained from the NIH HIV Reagent Program, Division of AIDS, NIAID, NIH, generously contributed by Dr. Thomas Folks. The cells were cultured in RPMI 1640 containing 2.0 mM of L-glutamine and 10% heat-inactivated fetal bovine serum (Lonza Bioscience, Morrisville, NC, USA) and maintained in the log phase with >98% viability before plating at 10^6^/mL in 12-well plates and stimulation. The cells were used with no more than five passages. Levels of p24 antigen were determined by ELISA (HIV-1 p24 ELISA assay, Xpress Bio, Frederick, MD, USA), according to the manufacturer’s instructions.

### 2.2 Treatments

Following optimization assays, DA hydrochloride (H8502, Sigma-Aldrich, St. Louis, MO, USA) was used at 1 and 10 μM (in p24 assays) and 1 μM in scRNAseq experiments, to mimic levels found in the brains of drug users (24). DRD1/DRD5 agonist SKF38393 hydrobromide (Tocris Bioscience) and DRD4 agonist PD168077 maleate (Sigma-Aldrich) were used at 10 μM. All treatments were performed for 24 h prior to the preparation of cells for sequencing. The latency reversal agent Ingenol-3-angelate (PEP005, Cayman Chemicals, Ann Harbor, MI) was used as a positive control for latency at 1 μM (**Supplementary Figure 1A**). The CD36 inhibitor sulfosuccinimidyl oleate sodium (SSO) was used at 20 μM. All control conditions were performed with DMSO to mimic stock dilutions. All treatments were performed in triplicates, in two independent experiments.

### 2.3 RT-PCR

Total RNA was extracted from samples using the Nucleospin RNA isolation kit (Macherey-Nagel, Allentown, PA) and cDNA was obtained using RT^2^ First-strand kit (Qiagen), according to the manufacturer’s instructions. SYBR Green real-time PCR was performed using RT^2^ PCR Primer sets for human DA receptor 1 (DRD1, GeneGlobe ID: PPH01857F), receptor 2 (DRD2, GeneGlobe ID: PPH01876C), receptor 3 (DRD3, GeneGlobe ID: PPH01894A), receptor 4 (DRD4, GeneGlobe ID: PPH10809A), and receptor 5 (DRD5, GeneGlobe ID: PPH00800F), all from Qiagen. Human GAPDH (GeneGlobe ID: PPH00150F, Qiagen) was used as housekeeping control. The expression was normalized to mRNA level of the housekeeping gene GAPDH.

### 2.4 Single-cell RNAseq samples preparation

10x Genomics Chromium Next GEM Single Cell 3’ Kit v3.1 (10x Genomics, Pleasanton, CA, USA) was used to prepare gene expression libraries for all samples. Following stimulation, cells were harvested by centrifugation (350× *g*, 5 min, room temperature) and washed with PBS containing 2% bovine serum albumin (BSA; Sigma-Aldrich). Approximately 10,000 viable cells were loaded onto a 10x Genomics Chip G along with barcoded gel beads and partitioning reagents using the Chromium Controller to generate gel bead-in-emulsion (GEM) droplets for single-cell mRNA capture. Library quality and quantity were assessed using the Agilent 2200 TapeStation System (Agilent, Santa Clara, CA, USA). Samples were sequenced on the Illumina NovaSeq 6000 platform, targeting a minimum sequencing depth of 20,000 read pairs per cell.

### 2.5 Single-cell RNAseq quality and data analysis

Sequencing data were processed using Cell Ranger 7.2.0 and visualized in the Loupe 7.0 browser (10x Genomics). For that, the data were demultiplexed and aligned to a customized hg38 *Homo sapiens* reference genome (NCBI RefSeq assembly GCF_000001405.40) combined with a chromosome representing the HIV-1 genome (AF033819) as previously described by us (1). The resulting sequencing libraries were then aggregated using the Cell Ranger Aggr (v3.1.0) function; low-quality single-cell transcriptomes were filtered based on UMI count (500 to 30,000), gene count (300 to 5,000), and mitochondrial percentage (less than 15%). **Table 1** shows the Cell Ranger-derived quality controls. A differential expression (DE) analysis was performed to identify signatures in each phenotypic cluster relative to the rest of the sample, giving an estimate of the log2 ratio of expression in a cluster to that in all other cells. The *p*-value of the expression difference is based on a negative binomial test adjusted for multiple testing via the Benjamini–Hochberg procedure. Features were filtered by mean UMI counts > 1.0, and the top N features by L2FC for each cluster were retained. Features with L2FC < 0 or adjusted *p*-value ≥ 0.10 were grayed out. The number of top features per cluster was recorded.

**Table 1:** Cell Ranger-derived quality assessment and count summary.

	Estimated number of cells	Mean reads per cell	Median genes per cell	Reads mapped to ref genome
NT	11,300	29,705	3,434	84.1%
DA	12,634	33,084	3,672	85.1%
PD168077	16,520	21,133	2,658	81.1%
SKF38393	14,146	29,786	3,501	85.4%

### 2.6 Data normalization, batch correction, and clustering analysis

Raw gene expression data in duplicates were processed and analyzed using a multi-step bioinformatic pipeline. Gene expression counts were first normalized using Seurat v4 (Satija Lab; https://satijalab.org), employing the default log-normalization approach to correct for differences in sequencing depth across cells and to render expression values comparable across the dataset. To account for technical variation introduced by differences in sample processing, sequencing batches, or donor-specific effects, batch correction was subsequently applied using Harmony, an algorithm designed to integrate single-cell datasets across multiple conditions while preserving genuine biological variation. Following normalization and batch correction, dimensionality reduction was performed within Seurat v4. Feature selection was restricted to the 2,000 most highly variable genes, identified using default parameters, to focus downstream analyses on the most biologically informative features. Principal component analysis (PCA) was then applied, and the 50 most statistically significant principal components were retained for downstream neighborhood graph construction. Low-dimensional visualization of the resulting cellular manifold was achieved using Uniform Manifold Approximation and Projection (UMAP), enabling intuitive two-dimensional representation of transcriptional similarities and differences among cell populations. Cluster visualizations and DE by cluster were performed in Loupe. Loupe was also used for the introduction of a gag-pol-nef-env > 0 filter in advanced selection, followed by a feature-based DE by cluster, and by stimulus, to identify similarities and differences in actively transcribing HIV genes between populations. Cluster-specific pathways and biological processes were analyzed using iPathwayGuide (Advaita Bioinformatics, Ann Arbor, MI, USA) (25), as well as the Database for Annotation, Visualization and Integrated Discovery (DAVID) (26), and visualized using GeneMania (27) in Cytoscape 3.10.2 (28, 29).

*Differential gene expression (DE) analysis* was conducted in duplicates, using the DESeq2 Package for R v3.21. Cell Ranger was integrated to calculate DGEs and generate volcano plots for visualization of differential gene expression upon different stimulations: non-treated (NT), DA, DRD1/DRD5 agonist SKF38393 (SKF), and DRD4 agonist PD168077 (PD). Statistical significance was tested using the Wilcoxon matched-pair signed-rank test with Benjamini–Hochberg corrections. Cell Ranger data were converted to Seurat for aggregation and cluster analysis. All raw and processed data were deposited in the Gene Expression Omnibus (GEO) with the assessment number GSE221531 (DRD agonists and antagonist experiments).

### 2.7 Pathway analysis, signatures, and visualizations

Each cluster was treated as an individual experiment for input in.txt format, used to identify significantly impacted signaling pathways, gene ontology terms, disease processes, predicted upstream regulators, and putative mechanisms based on significant gene expression signatures. Comparisons between clusters of interest were performed using meta-report features in iPathwayGuide (Advaita Bioinformatics, Ann Arbor, MI). Gene network analysis was performed in Cytoscape v3.10.2 (29), with GeneMania plugin (30, 31), and *Homo sapiens* sources from BioGRID_ORGANISM (32–34). Permutation analysis in iPathwayGuide was used to rank gene lists by cluster matched to the Gene Set Enrichment Analysis (GSEA) database (35, 36). Upstream regulator prediction was performed using TWP v2.0 in iPathwayGuide platform.

## 3 Results

### 3.1 DRDs and latency reversal

**Figure 1:**
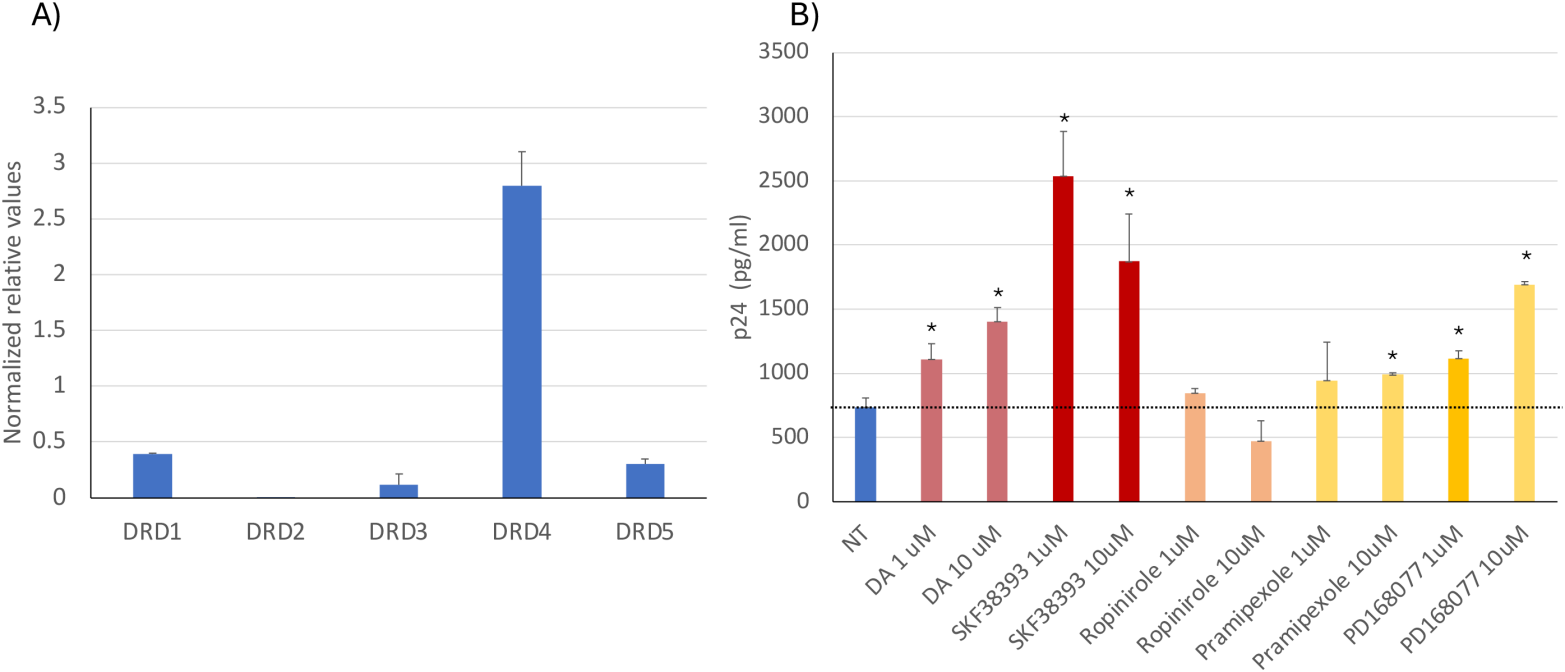
DRD expression and effect of DA via its receptors on HIV p24 levels in U1 cells. **(A)** DRD relative values in unstimulated U1 cells, tested by RT-PCR, normalized to the housekeeping gene GAPDH. **(B)** HIV p24 was measured in U1 supernatants 24 h after stimulation with DA (1 and 10 μM), or with selective agonists SKF38393 (DRD1–DRD5), Ropinirole (DRD2), Pramipexole (DRD3), and PD168077 (DRD4), by ELISA. Values of three independent experiments performed in duplicate were normalized to cell numbers in each experiment and correspond to the mean ± SD. A punctuated line indicates non-treated (NT, vehicle) control levels. *p < 0.05 in comparison to NT.

This study seeks to understand the common pathways associated with latency reversal by DA using U1 cells as an *in vitro* model of HIV latency in promonocytes. U1 cells expressed higher levels of DRD4, followed by lower levels of the Type I DA receptors, DRD1 and DRD5. DRD2 was not detectable and DRD3 was not consistently detected (**Figure 1A**). Latency of U1 cells was confirmed by the use of PEP005 (**Supplementary Figure 1A**). Reversal was also observed in 10%–15% of the U1 cells (calculated based on the total amount of p24 elicited by PEP005), in conditions of exposure to the neurotransmitter DA, as well as by the selective pharmacological activation of a type I DRD, predominantly DRD1 by SKF38393, or the type II receptor DRD4 by PD168077 (**Figure 1B**). Ropinirole, a DRD2 agonist, did not affect levels of p24, and the DRD3 agonist Pramipexole increased p24 only at a higher dose. We thus focused on DRD1 and DRD4 effects, due to the relative abundance and the effects of selective agonists on p24.

### 3.2 DA, DRD1, and DRD4 signatures

**Figure 2:**
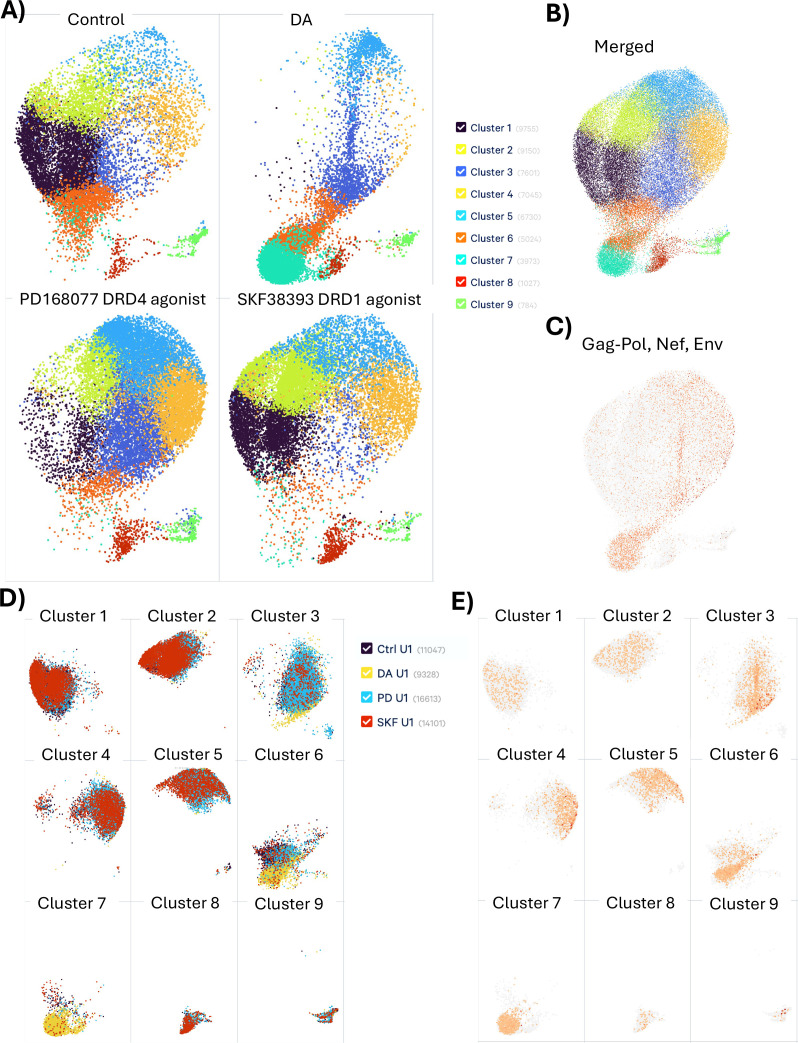
Visualization of cellular clusters resulting from DA, DRD1, and DRD4 selective stimulation, and HIV gene expression. UMAP plots of transcriptomic data in single-cell RNAseq datasets from two independent experiments. U1 cells were cultured with vehicle, 1 μM DA, 10 μM SKF38393, or 10 μM PD168077 for 24 h. Cells were collected and processed for characterization using single-cell RNAseq. Cluster analysis and visualization were performed: **(A)** UMAP for dimension reduction of data in individual treatment conditions; **(B)** merged treatment conditions with color-coded clusters; and **(C)** merged clusters with orange dots indicating events positive to HIV Gag-Pol-Env genes in U1 cell populations. **(D)** Individual clusters color-coded for identification of stimulant conditions and **(E)** parallel HIV genes Gag-Pol-Env positively transcribing events in orange.

The single-cell approach proved critical to select and characterize cells that actively transcribed HIV genes under the conditions of interest as previously described by us (1), and to facilitate the determination of common as well as unique signatures linked to latency reversal caused by neurotransmitters associated with substance use disorders, via receptor binding. We focused on DRD1 and DRD4 stimulation, which caused significant p24 increase. **Figure 2** shows the dynamic cellular cluster subpopulations occurring in control non-treated U1 cells, DA-stimulated cells, and cells selectively stimulated with the DRD4 agonist PD168077 or with the DRD1 agonist SKF38393 (**Figure 2A**). The merged nine clusters (**Figure 2B**) can be mapped to subsets with a higher incidence of cells transcribing HIV genes (**Figure 2C**). The individual clusters and how they relate to stimulants and latency reversal can be appreciated in parallel in **Figures 2D, E**.

**Figure 3:**
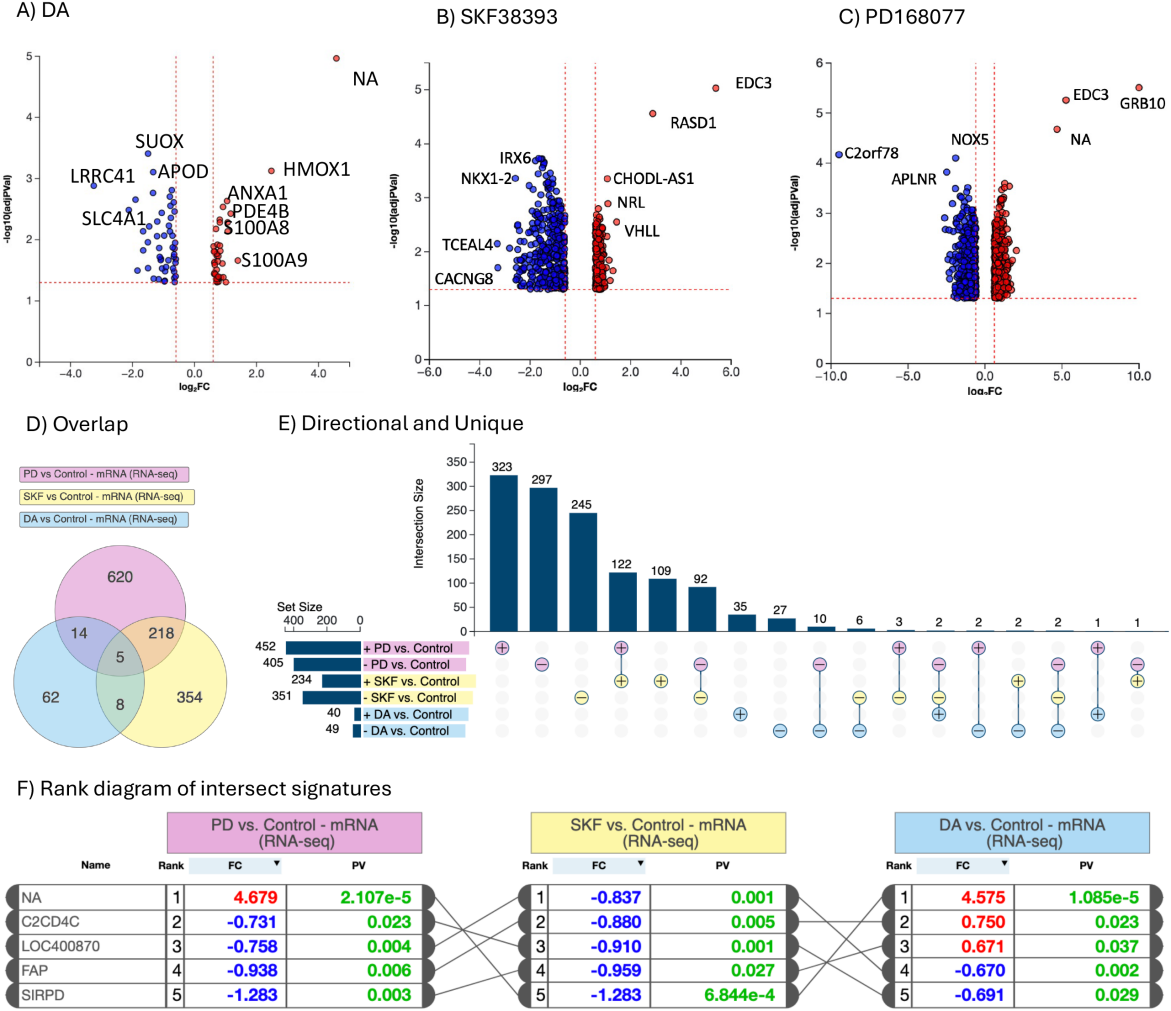
Signatures of DA, SKF38393, and PD168077. Volcano plots showing up- and downregulated genes by **(A)** DA, **(B)** DRD1 selective agonist SKF38393, and **(C)** DRD4 selective agonist PD168077. **(D)** Venn diagram showing the number of unique gene signatures, as well as intersects. **(E)** Upset plot indicating the number of genes changed by indicated conditions, showing directionality (+ and −) of unique and intersect signatures. **(F)** Rank diagram of five intersecting genes.

The analysis of transcriptional profiles showed strong differentially expressed genes out of 18,994 with measured expression using input thresholds of *p* < 0.05 for statistical significance. Interestingly, the meta-data analysis revealed that the number of genes modified by DA (89 genes) was smaller than that modified by the selective agonists SKF38393 (585 genes) or PD168077(857 genes), suggesting that the receptors may cross-regulate each other when simultaneously triggered, and regulatory elements may be not available in selective stimulations (**Figure 3**). These data were analyzed in the context of the Kyoto Encyclopedia of Genes and Genomes (KEGG) (37) updated in January 2025, and gene ontology from the Gene Ontology (GO) Consortium database (38, 39), using Bonferroni corrections for multiple comparisons. The signatures of stimulation with DA or with the selective agonists were unique, where genes with strong fold change (FC) caused by DA were not often among the strongest up- and downregulated genes in cells stimulated with the selective DRD agonists (**Figure 3**), although all of them triggered a significant increase in HIV gene transcription (**Figures 1**, **2**). Only five genes were altered by all three conditions, although for the most part with different directionality (**Figure 3E**). For instance, NA (neuroacanthocytosis, also known as X-linked Kx blood group antigen, Kell, and VPS13A binding protein) was 4.757-fold upregulated by DA (*p* = 1.085E-5) and 4.679-fold upregulated by the DRD4 selective agonist PD168077 (*p* = 2.107E-5), but downregulated by SKF38393 (1.283-fold, *p* = 6.844E-4). Other examples including the fibroblast activating protein alpha (FAP) and the signal regulatory protein delta (SIRPD) were both significantly upregulated by DA, but downregulated by both SKF38393 and PD. Two genes were significantly downregulated by all three conditions: the C2 calcium-dependent domain containing 4C (C2CD4C) and an uncharacterized locus LOC400870. On the other hand, unique and partial intersect signatures were more frequent. For instance, EDC3 (enhancer of mRNA decapping 3) was significantly upregulated by both selective agonists (5.388-fold by SKF38393, *p* = 9.306E-6; 5.261 by PD168077, *p* = 5.526E-6), but not significantly changed by DA. **Table 1** shows genes expressed in response to DA and with significant change shared by either both or one of the selective agonists. Interestingly, we identified S100A8 and S100A9, which we previously described as genes encoding a complex mediator of viral transcription by DA, MRP8/14 (calprotectin) via the AGE/RAGE pathway etc. (2). Indeed, S100A8 and S100A9 were significantly increased by DA in this experiment (*p* = 0.007 and 0.022, respectively), but surprisingly not significantly affected by SKF38393 or PD168077alone.

While annotations to biological processes derived from DA stimulation resulted in inflammatory responses and chemotaxis (*p* = 0.02), host–virus interactions (*p* = 0.03), olfaction (*p* = 0.04), and anion exchange (*p* = 0.04), the stimulation with SKF38393 triggered processes in neurogenesis (*p* = 0.002), ion transport (*p* = 0.002), sensory transduction (*p* = 0.002), angiogenesis (*p* = 0.003), anion exchange (*p* = 0.03), oxygen transport (*p* = 0.04), differentiation (*p* = 0.04), and neurotransmitter transport (*p* = 0.05). The stimulation with PD168077, which showed higher cluster similarities with DA, had multicellular processes (*p* = 0.007), nervous system development (*p* = 0.005), host–virus interactions (*p* = 0.01), and G protein-coupled receptors (G-PCRs) (*p* = 0.029) as significant biological processes.

The specific genes that were shared between DA and PD168077or DA and SKF38393 (**Table 1**) were assigned to olfaction (*p* = 0.015), anion exchange (*p* = 0.015), sensory transduction (*p* = 0.02), G-PCR (*p* = 0.029), and virus–host interactions (*p* = 0.013). DRDs are GPCRs (40). Interestingly, DA and PD168077 shared signatures in virus–host interaction processes, and a significant decrease in genes such as SUPT16H, with functions in chromatin regulation, and a described role in maintaining HIV latency (41). SKF, on the other hand, shared fewer signatures with DA (**Table 2**).

**Table 2:** Fold change in relation to vehicle-stimulated controls of DA signatures overlapping with SKF38393 and/or PD198077, and their *p*-values.

Gene symbol	Entrez ID	Gene name	DA vs. control	*p*-value	SKF38393 vs. control	*p*-value	PD168077 vs. control	*p*-value
ADAM21	8747	ADAM metallopeptidase domain 21	-0.645	0.014	−0.73	0.01	−0.552	0.067
APOD	347	Apolipoprotein D	−1.334	7.84E−04	−0.917	0.202	−1.332	5.87E−04
ASB18	401036	Ankyrin repeat and SOCS box containing 18	−0.764	0.004	−0.685	0.02	-0.552	0.118
C2CD4C	126567	C2 calcium-dependent domain containing 4C	−0.691	0.029	−0.91	0.001	−0.731	0.023
CCL2	6347	C-C motif chemokine ligand 2	0.815	0.005	−0.205	0.41	−1.177	0.018
CHODL-AS1	54075	Long intergenic non-protein coding RNA 3147	−0.732	0.002	1.077	4.45E−04	1.217	0.119
FAP	2191	Fibroblast activation protein alpha	0.75	0.023	−0.88	0.005	−0.938	0.006
FGF19	9965	Fibroblast growth factor 19	−0.626	0.011	0.771	0.002	1.018	0.13
LOC400870	400870	Uncharacterized	−0.67	0.002	−0.837	0.001	−0.758	0.004
MARVELD3	91862	MARVEL domain containing 3	0.706	0.048	0.862	0.083	0.638	0.015
MZB1	51237	Marginal zone B and B1 cell specific protein	−0.958	0.047	0.044	0.946	1.434	0.024
NA	4663	Neuroacanthocytosis	4.575	1.09E−05	−1.283	6.84E−04	4.679	2.11E−05
OR13C9	286362	Olfactory receptor family 13 subfamily C member 9	−0.615	0.024	−0.61	0.25	−1.089	0.004
OR2T27	403239	Olfactory receptor family 2 subfamily T member 27	−0.713	0.009	−0.609	0.263	−1.114	0.002
OR52E4	390081	Olfactory receptor family 52 subfamily E member 4	−1.904	0.002	−1.519	0.26	−1.941	0.03
OR8K5	219453	Olfactory receptor family 8 subfamily K member 5	−0.668	0.019	−0.73	0.01	−0.564	0.072
PAQR4	124222	Progesting and adipoQ receptor family member 4	−0.779	0.003	−0.718	0.005	−0.572	0.088
PLEKHA5	54477	Pleckstrin homology domain containing A5	−1.048	0.03	−0.557	0.387	−1.133	0.023
RASGRF1	5923	Ras protein specific guanine nucleotide releasing factor 1	−1.842	0.032	−1.932	0.217	−2.397	0.008
RORA	6095	Retinoic acid receptor related orphan receptor A	−1.086	0.02	0.841	0.054	1.635	0.01
SIRPD	128646	Signal regulatory protein delta	0.671	0.037	−0.959	0.027	−1.283	0.003
SLC4A1	6521	Solute carrier family 4 member 1	−1.659	0.007	−1.217	0.252	−1.811	0.019
SLC4A8	9498	Solute carrier family 4 member 8	−0.706	0.013	−1.238	6.31E−04	−0.669	0.287
SLCO1A2	6579	Solute carrier organic anion transporter family member 1A2	−1.656	0.015	−0.961	0.337	−1.915	0.005
SUPT16H	11198	SPT16 homolog, facilitates chromatin remodeling subunit	−0.605	0.021	−0.371	0.074	−0.608	0.019
THAP10	56906	THAP domain containing 10	−0.611	0.04	−1.206	0.001	−0.63	0.101
TMEM54	113452	Transmembrane protein 54	−1.203	0.005	−1.169	0.289	−1.644	0.002

Up- and downregulated genes are indicated in red and blue, respectively. Significant *p*-values are highlighted in green.

Overall, this suggests that the upregulation of viral transcription by DA is replicated by DRD1- or DRD4-selective agonists, but with unique gene signatures and for potentially different reasons. An in-depth characterization of cluster characteristics using the single-cell approach that allows identification of HIV-positive transcription may pinpoint commonalities in pathways and biological processes by the DA receptors showing relevance for latency reversal.

### 3.3 Phenotypic clusters characteristics

**Figure 4:**
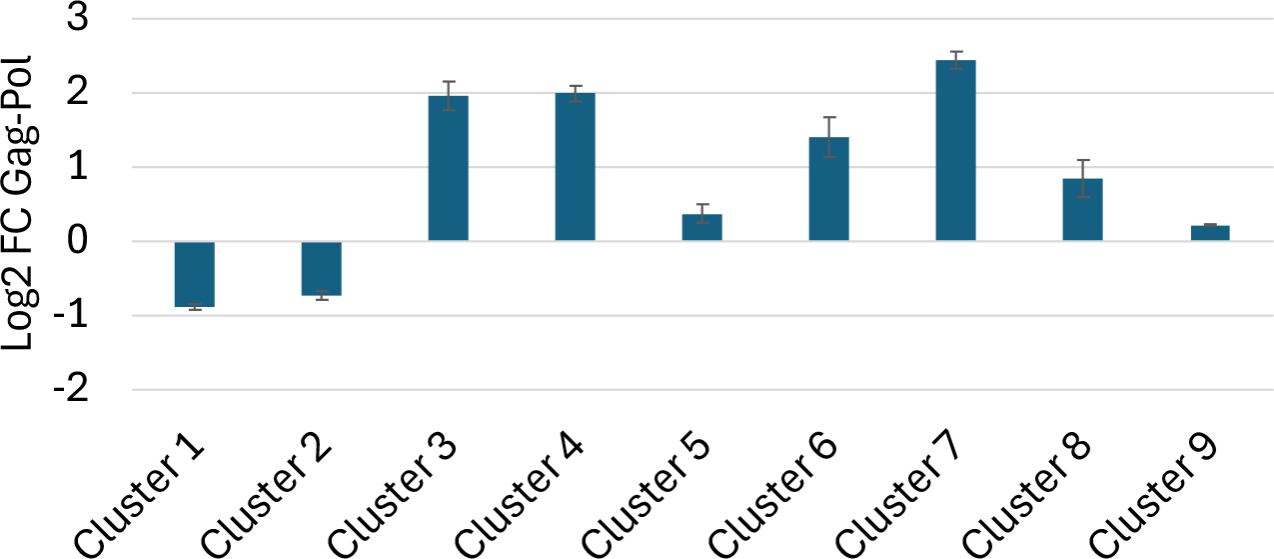
Average active transcription of HIV Gag-Pol in single-cell clusters. The relative expression (log2 fold change – FC) of HIV Gag-Pol within each specific cluster compared to all other cells. Positive values indicate gene upregulation and negative values indicate downregulation in the assigned cluster.

The identification of clusters using the single-cell approach was instrumental to identify profiles that were more likely linked to increased HIV gene transcription by DA or by the selective agonists of DRD1 and DRD4. **Table 3** shows the summary of clusters. We grouped them arbitrarily by comparing their distribution in relation to upregulated HIV genes by cluster (**Figure 4**) and by their presence or absence in each condition.

**Table 3:** Summary of cluster characteristics, with associated conditions, HIV gag-pol relative expression, biological processes and pathway assignments, and predicted activated upstream regulators identified by iPathwayGuide.

Clusters	Condition	HIV Gag-Pol log2FC	Biological processes	Pathways	Activated upstream regulators predicted**
1	Ctr, PD, SKF	−0.8991825*	Host–virus interaction; apoptosis; cell adhesion; growth regulation	Sphingolipid signaling; focal adhesion	KDM6A; CTNNB1; TGFb1; SMAD2; IL10
2	Ctr, PD, SKF	-0.737568*	No assignment	No assignment	GPSM1; GPSM2; GPSM3; PCP2; IL4
3	Ctr, DA, PD, SKF	1.95723201*	Cell adhesion	Malaria	JAG1; ALK; CCNC; NELL1
4	Ctr, PD, SKF	1.98324364*	Immune response; inflammation; chemotaxis; autophagy	Innate immune system; AGE-RAGE signaling	ANXA1
5	Ctr, DA, PD, SKF	0.36230224	Respiratory chain; host–virus interaction; phagocytosis	Neurodegenerative diseases; oxidative phosphorylation	NANOG; CREBBP; TP53
6	Ctr, DA, PD, SKF	1.39686912	Apoptosis	Diabetic cardiomyopathy; thermogenesis	NFKAPPAB65; CEBPB; LHX3
7	DA	2.42857119*	Innate immune response; superoxide metabolic process; positive upregulation of IL1beta	Asthma; natural killer cell toxicity; lipids and atherosclerosis	NCOA3; MED1; NCOA1; NCOA1
8	Ctr, DA, PD, SKF	0.84179792	Respiratory chain	Neurodegenerative diseases; oxidative phosphorylation	AIMP1; SEPTIN2
9	Ctr, DA, PD, SKF	0.21271164	No assignment	PPAR signaling; leukocyte transmigration	CD9; GCM1; AIMP1

*p < 0.05, ** displayed the four most significant regulators.

### 3.4 Phenotypic clusters linked to positive HIV gene transcription

**Figure 5:**
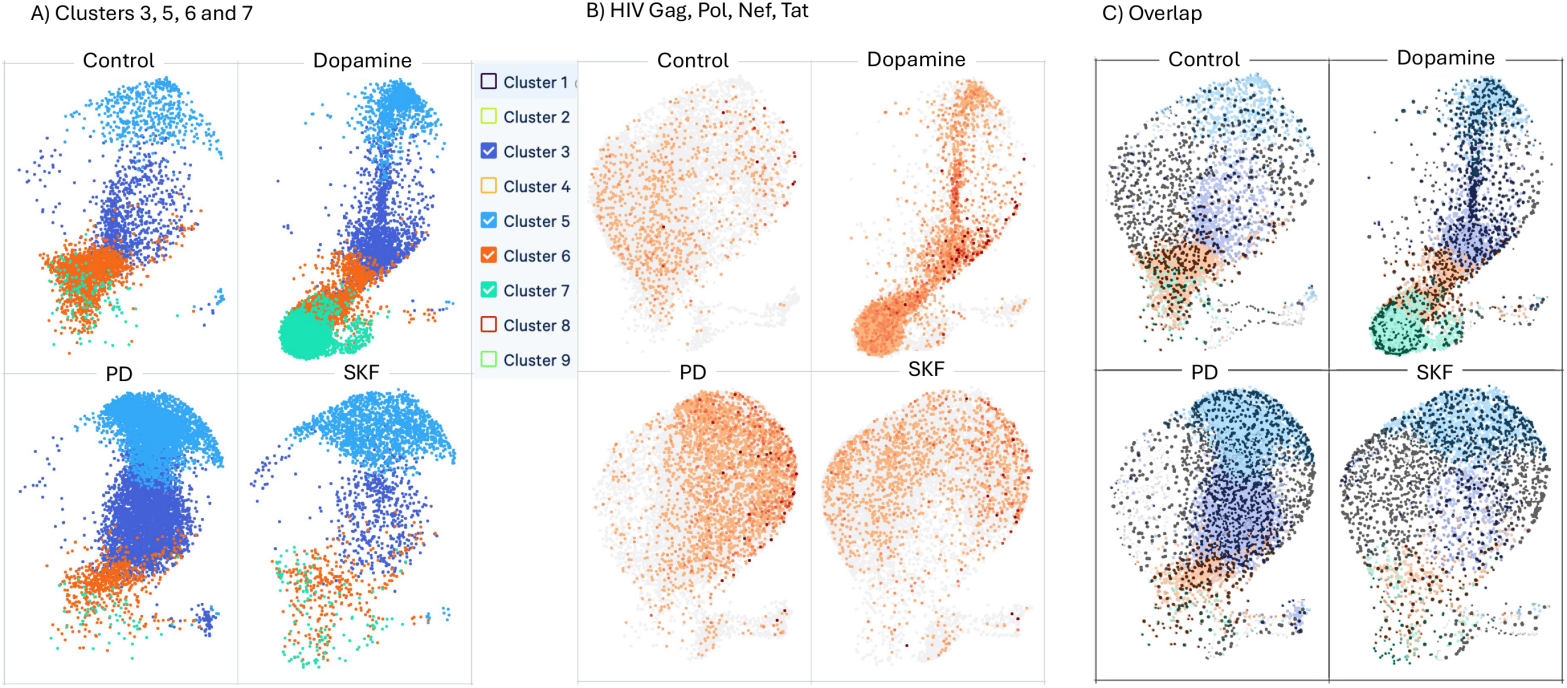
Mapping of clusters with the highest HIV gene transcription following treatment with DA, DRD1, and DRD4 selective stimulations. **(A)** Mapping of clusters 3 (dark blue), 5 (light blue), 6 (orange), and 7 (green) in experimental conditions (Control, DA, SKF38393—SKF, and PD168077—PD). **(B)** Events representing cells with active HIV transcription (shades of orange indicate level in relation to average—up to fivefold). **(C)** Overlap between clusters and HIV-positive events (black dots).

First, we focused on clusters with higher HIV gene transcription. These clusters were 3, 5, and 6 represented in cells stimulated with DA and in DRD1- and DRD4-selective stimulations and showing associated HIV gene upregulation (**Figures 2**, **4**) to look for gene signatures, pathways, and biological processes that are common. We also focused on cluster 7 that was exclusively triggered in DA stimulation with strong HIV gene transcription (**Figures 2**, **4**). **Figure 5** shows these clusters (**Figure 5A**), in relation to the expression of HIV genes (**Figure 5B**), and their overlap in all conditions (**Figure 5C**). The mapping of clusters and HIV+ events suggests that DA may have commonalities with PD198077, and that cluster populations that trigger virus transcription in SKF38393 stimulation may do it so in populations with distinct signatures but potentially overlapping pathways.

**Figure 6:**
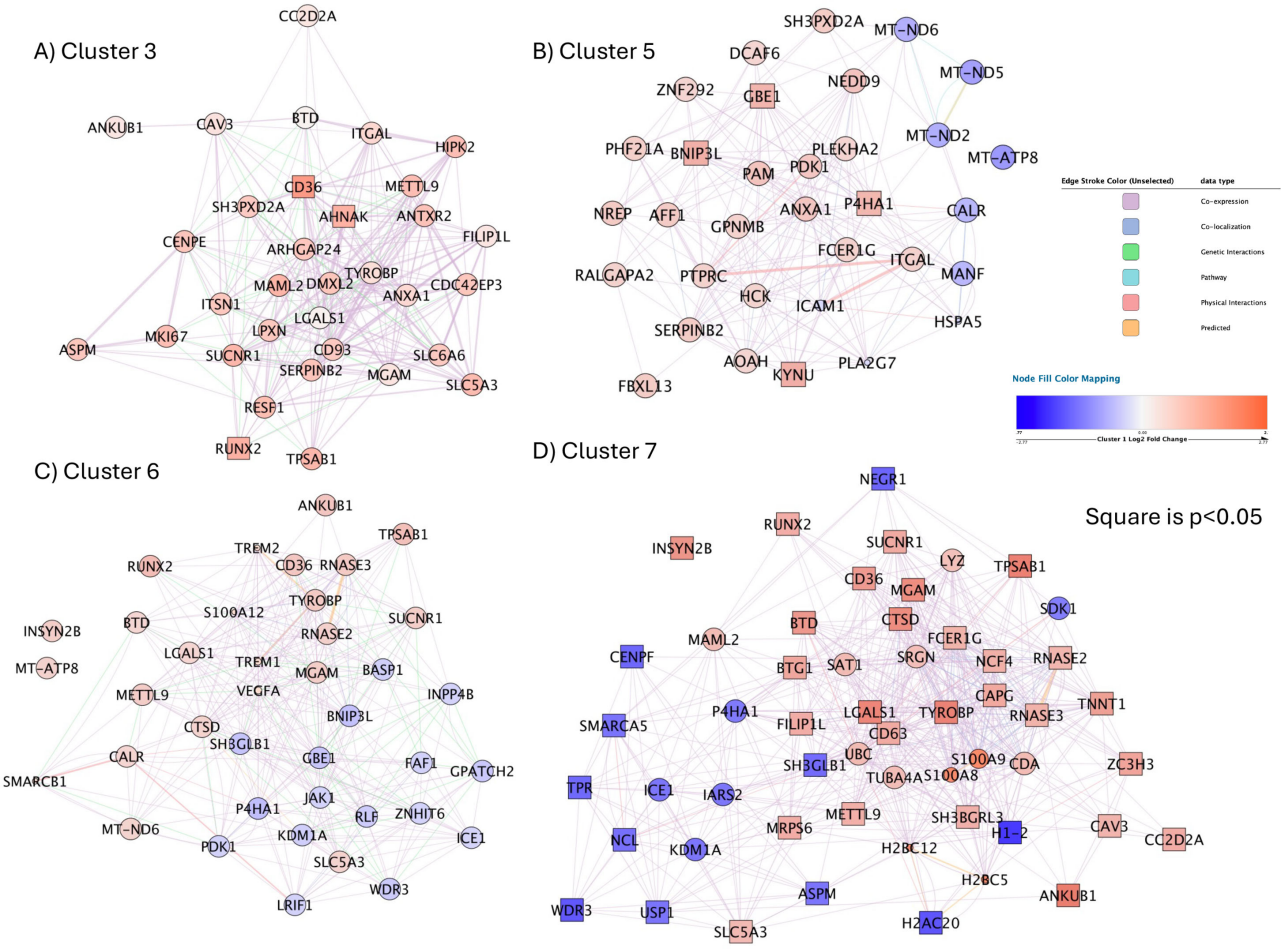
Gene network analysis of clusters 3, 5, 6, and 7 upregulated by DA and exhibiting upregulation of HIV transcripts. Gene network visualization for **(A)** cluster 3 expressed by all conditions, **(B)** cluster 5 expressed by all conditions, **(C)** cluster 6 expressed by all conditions, and **(D)** cluster 7 expressed only by DA stimulation. Nodes represent genes expressed in assigned clusters. Shades of red indicate levels of upregulation and shades of blue represent downregulation (−3.5- to 3.5-fold). Square shapes indicate significance (*p* < 0.05). Genes are connected based on pathway (light red connectors), genetic interactions (green connectors), co-expression (pink connectors), and physical interactions (blue connectors).

**Figure 7:**
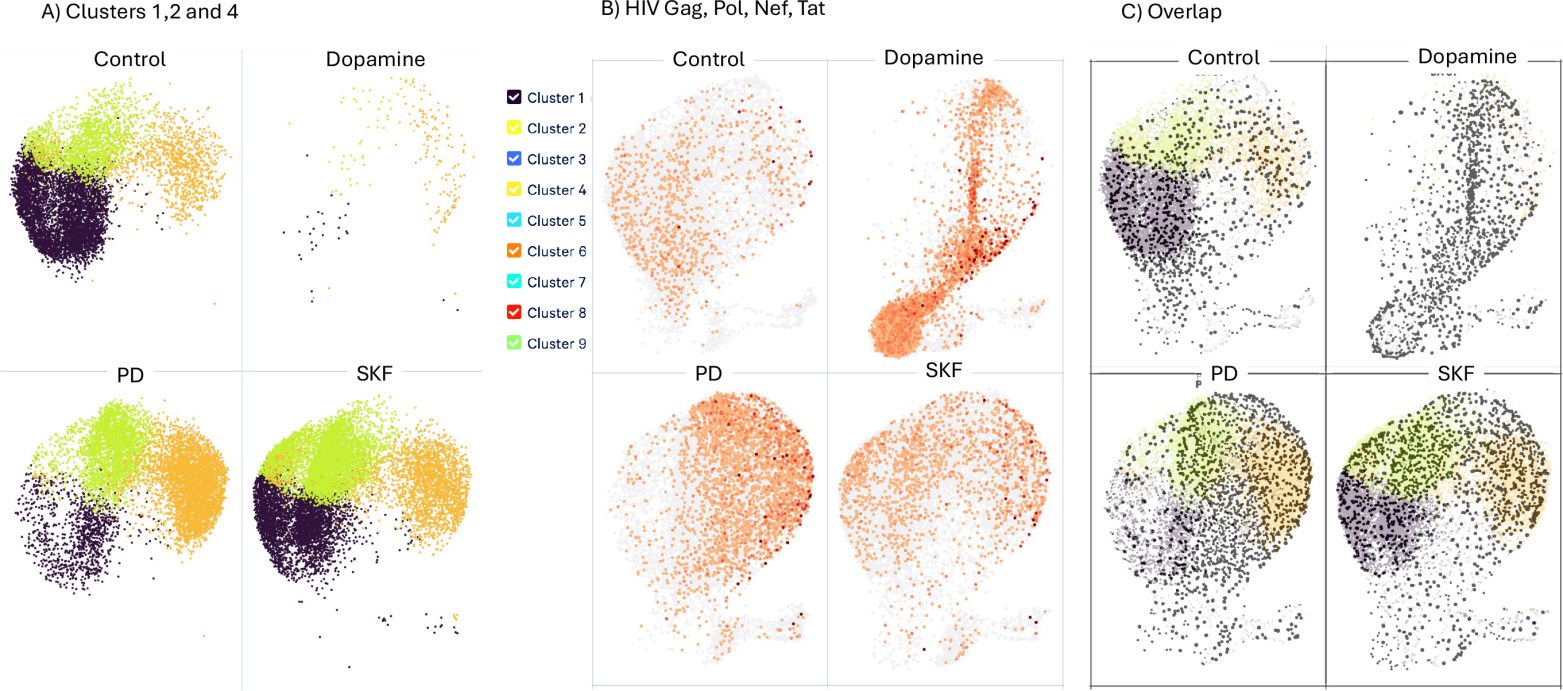
Mapping of clusters with increased HIV gene transcription following DRD1 and DRD4 selective stimulations, but suppressed by DA. **(A)** Mapping of clusters 1 (black), 2 (light green), and 4 (light orange) in experimental conditions (Control, DA, SKF38393—SKF, and PD168077—PD). **(B)** Events representing cells with active HIV transcription (shades of orange indicate level in relation to average—up to fivefold). **(C)** Overlap between clusters and HIV-positive events (black dots).

While clusters 3, 5, and 6 are mostly shared between DA and PD, but also present in SKF38393 stimulation, cluster 7 is strongly and exclusively assigned to DA (**Figure 5**). Clusters 3, 6, and, expectedly, 7 had a more robust above average expression of HIV gene transcripts (**Figure 4**), while cluster 5 had just a modest non-significant increase. Gene network analysis indicated that clusters with HIV-positive transcripts had orchestrated patterns but little or no overlapping signatures. An exception was CD36 upregulation, which was significant in both clusters 3 (**Figure 6A**) and 7 (**Figure 6D**), and substantial in cluster 6 (**Figure 6C**).

Along with the strongest HIV gene transcription, cluster 7 contained the strongest DA-mediated upregulated signatures such as S100A8 and S100A9 (encoding the MRP8/14 protein complex), decreased genes of structural constituents of chromatin such as H2AC20 and H1-2, but also increased RUNX2 and ZC3H3, which enable DNA binding activity (**Figure 6D**). Altogether, cluster 7 signatures annotate to biological processes of innate immune response (*p* = 0.039), superoxide metabolic process (*p* = 0.03), and positive upregulation of IL1beta (*p* = 0.024), and pathways in asthma (*p* = 0.002), natural killer cell toxicity (*p* = 0.007), and lipids and atherosclerosis (*p* = 0.051).

Cluster 5, which marginally affected HIV transcription, shows decreased expression of mitochondrial respiratory chain genes (MT-ND2, 5, and 6) and mitochondrial ATP synthase 8 (MT-ATP8), with links to pathways in neurodegenerative diseases (*p* = 0.05) (**Figure 6B**).

### 3.5 Clusters linked to selective DRD stimulation

SKF38393 had a strong representation of clusters 1, 2, and 4, all suppressed in DA stimulation (**Figure 7**). Increased active HIV transcripts were detected in cluster 4, which was enriched by SKF38393 and PD168077compared to controls (**Figures 4**, **7**). Clusters 1 and 2 had active HIV transcripts but below the average transcription (**Figure 4**). Cluster 4, on the other hand, had higher HIV gene transcription in DRD1 stimulation, shared with PD (**Figures 4**, **7**). Importantly, clusters 1, 2, and 4 were suppressed by DA stimulation (**Figures 2**, **7**).

**Figure 8:**
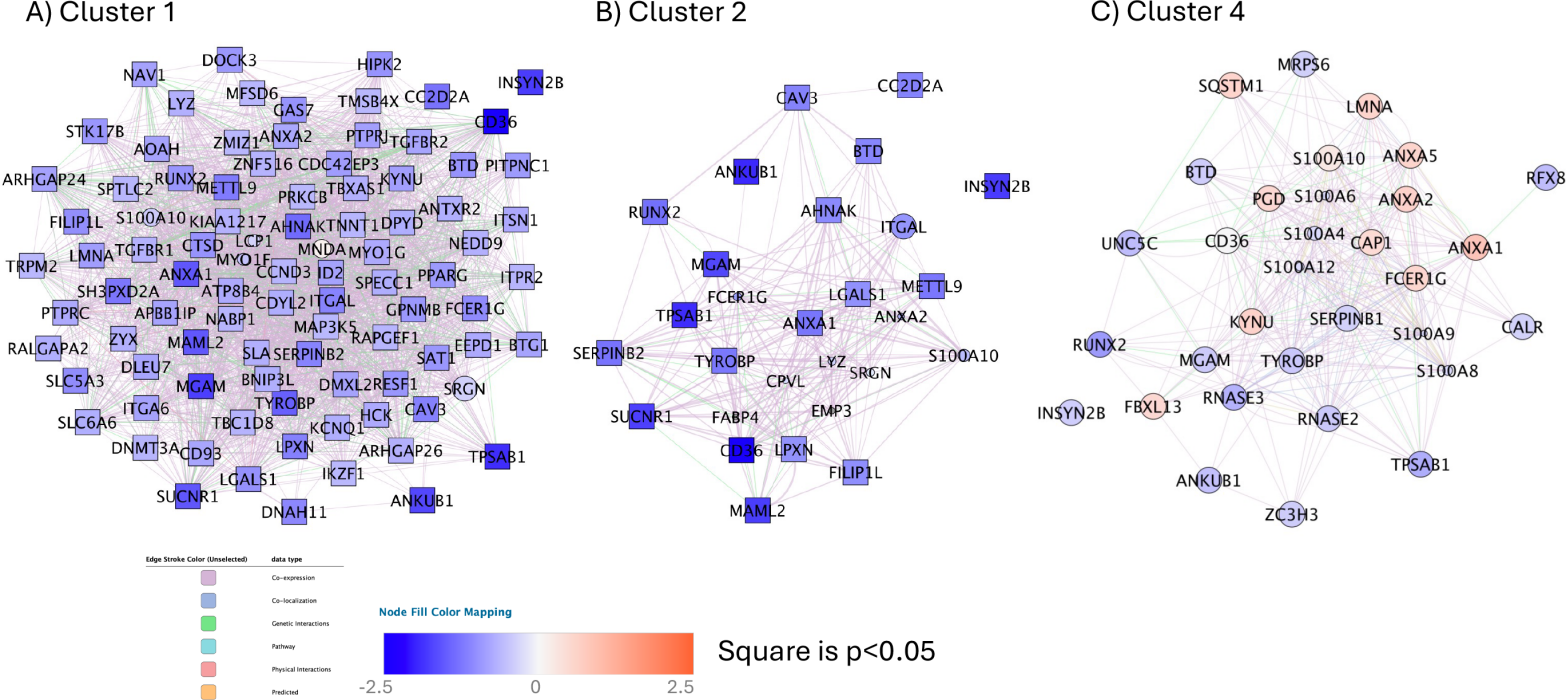
Gene network analysis of clusters 1, 2, and 4, suppressed by DA. Gene network visualization of **(A)** cluster 1, **(B)** cluster 2, and **(C)** cluster 4, all expressed by Ctr, PD168077, and SKF38393 but not by DA. Nodes represent genes expressed in assigned clusters. Shades of red indicate levels of upregulation and shades of blue represent downregulation (−2.5- to 2.5-fold). Square shapes indicate significance (*p* < 0.05). Genes are connected based on pathway (light red connectors), genetic interactions (green connectors), co-expression (pink connectors), and physical interactions (blue connectors).

Gene network analysis of clusters linked to SKF38393 indicated that clusters 1 and 2 are mainly characterized by strong orchestrated gene downregulation (**Figures 8A, B**). In cluster 1, the downregulated gene network was annotated to biological processes in host–virus interaction (*p* = 0.011), apoptosis (*p* = 0.011), cell adhesion (*p* = 0.03), and growth regulation (*p* = 0.03). Pathways associated with this pattern were sphingolipid signaling (*p* = 0.049) and focal adhesion (*p* = 0.049). Cluster 2 did not align with biological processes, and despite the fact that genes in natural killer cell-mediated cytotoxicity were represented, the pathway assignment was not significant. Interestingly, in contrast to clusters with enhanced HIV transcription, CD36 was one marker with the power of distinguishing latency by being downregulated in these clusters with below average HIV gene transcription. Cluster 4, on the other hand, had a significant representation of immune response genes (*p* = 0.0007) and inflammation (*p* = 0.001) in biological processes and the AGE-RAGE signaling pathway (*p* = 0.006) with several calcium binding components represented, including S100A8 and S100A9, in alignment with positive HIV gene transcription (**Figure 8C**).

The similarities between clusters and total transcriptome profiles by stimulant conditions leading to HIV gene transcription may offer insights into the role of DA via its receptors in latency reversal, the role of receptor signaling, and the different underlying mechanisms that can trigger HIV gene transcription from integrated latent virus. We previously showed that S100A8 and S100A9 (MRP8/14 calprotectin) are a DA signature that can reverse latency via RAGE (2).

### 3.6 Signatures and pathways triggered via DRDs

Given the consistent identification of CD36 upregulation in clusters with positive HIV transcription, we considered that this marker may be involved in latency. Under the hypothesis that the blockage of CD36 would prevent the DA-induced increase in p24, we incubated the U1 cells with a pharmacological blocker (SSO) 30 min prior to DA exposure. However, the blocker of CD36 alone increased p24 and did not prevent the effects of DA (**Supplementary Figure 1B**).

**Figure 9:**
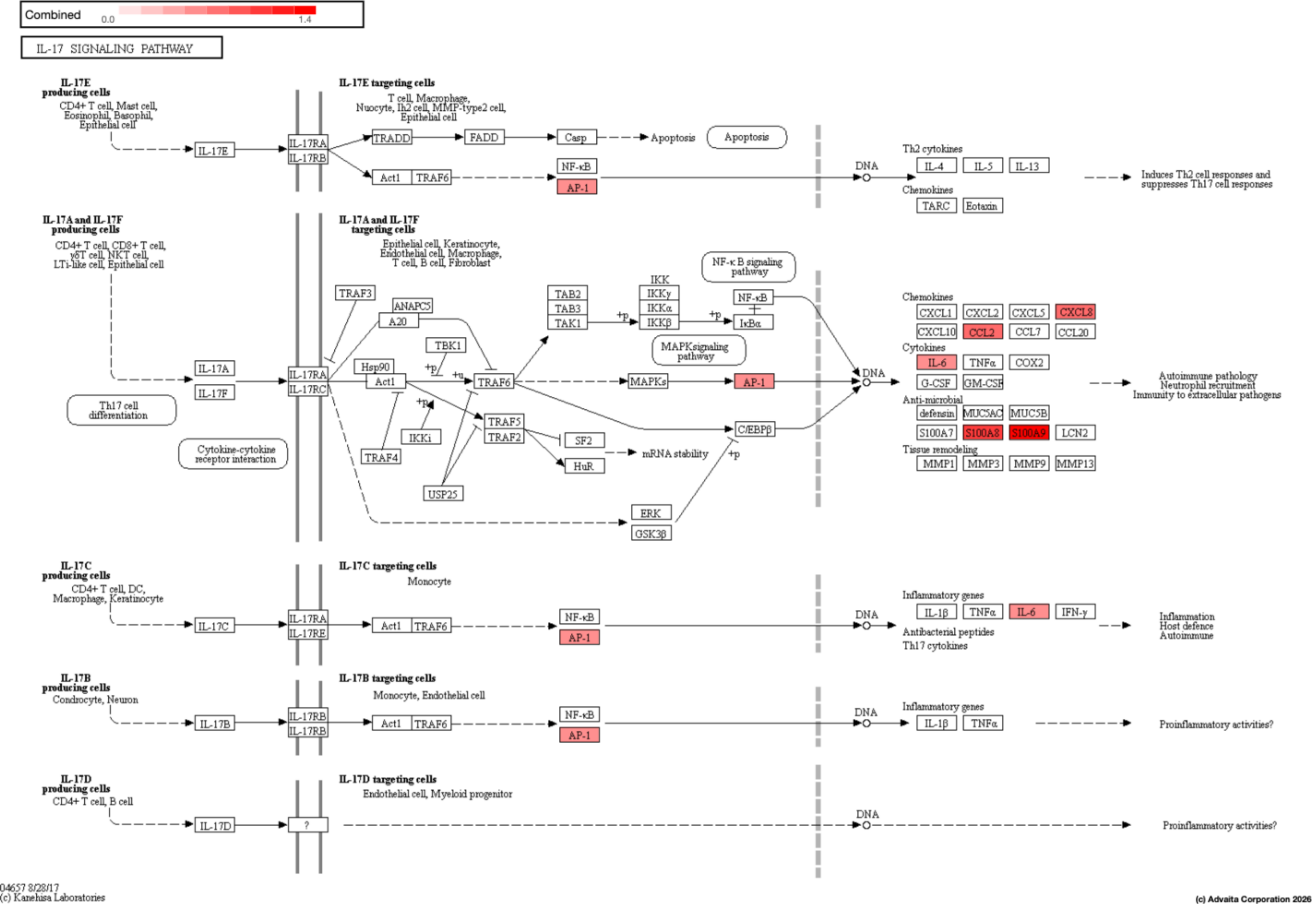
Diagram representation of the top most significant pathway perturbed by stimulation with the DA. DA-induced Th17 signaling pathway was significantly enriched. The pathway diagram is overlaid with the computed perturbation of each gene, accounting for both the gene fold change and for the accumulated perturbation propagated by any upstream genes. The highest negative perturbation is shown in dark blue, while the highest positive perturbation is shown in dark red. The legend describes the value on the gradient.

**Figure 10:**
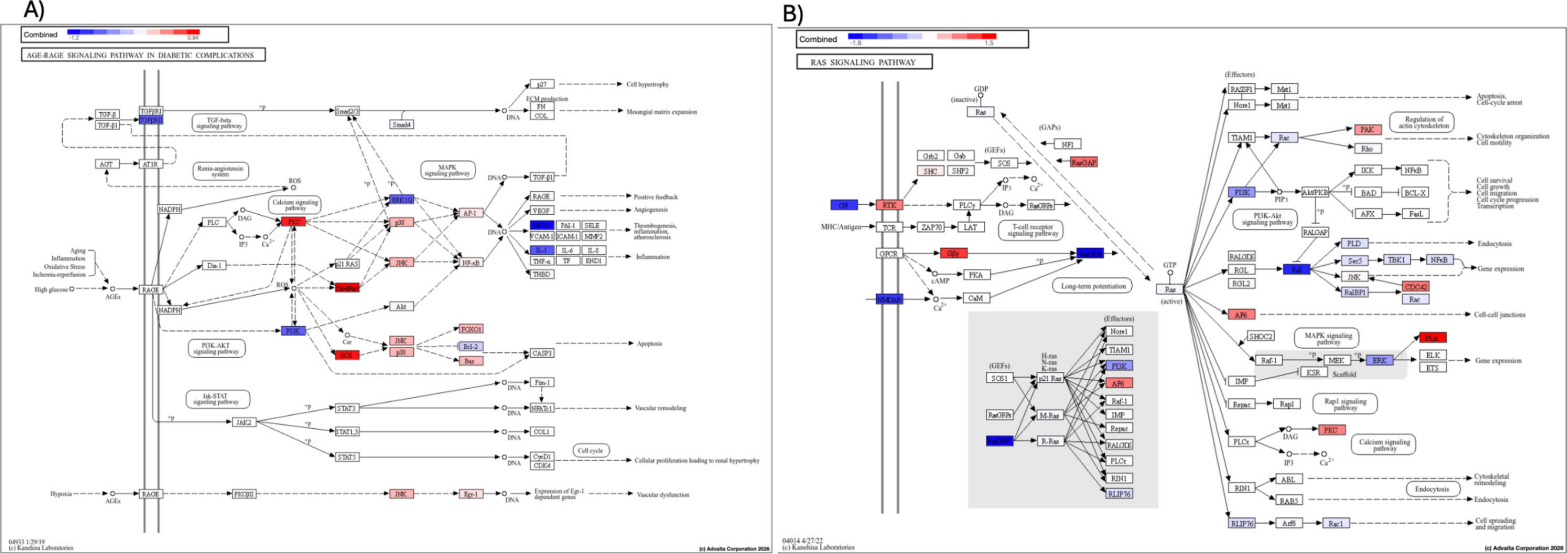
Diagram representation of virus and inflammation-relevant pathways significantly perturbed by stimulation with PD168077—PD. The pathway diagrams are overlaid with the computed perturbation of each gene, accounting for both the gene fold change and for the accumulated perturbation propagated by any upstream genes. The highest negative perturbation is shown in dark blue, while the highest positive perturbation is shown in dark red. The legend embedded in each pathway described the value on the gradient. The pathways represented have been selected among the top five pathways for each stimulation due to their potential links with inflammation and viral control. **(A)** DRD4-induced AGE-RAGE signaling in diabetic complications, and **(B)** DRD4-induced Ras signaling pathway.

**Figure 11:**
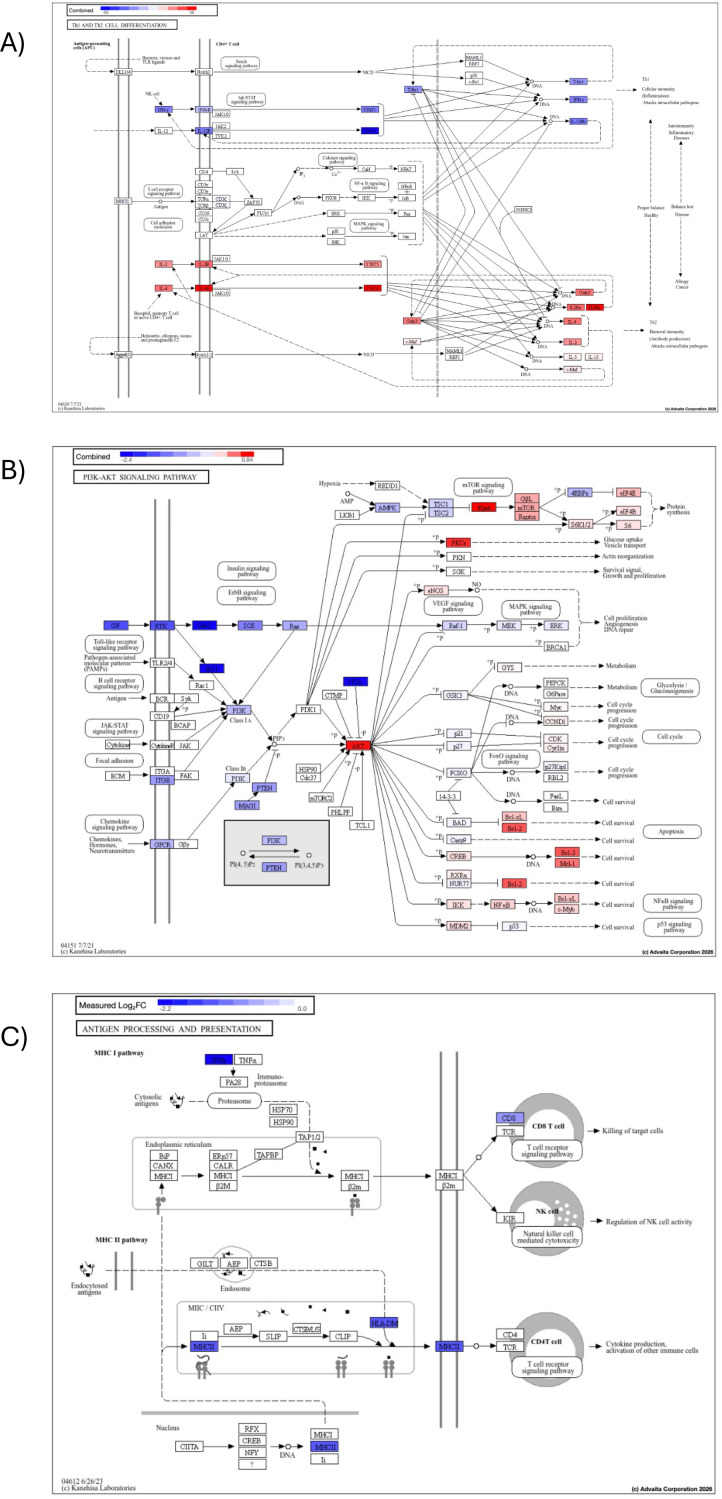
Diagram representation of virus and inflammation-relevant pathways significantly perturbed by stimulation with SKF38393—SKF. The pathway diagrams are overlaid with the computed perturbation of each gene, accounting for both the gene fold change and for the accumulated perturbation propagated by any upstream genes. The highest negative perturbation is shown in dark blue, while the highest positive perturbation is shown in dark red. The legend embedded in each pathway described the value on the gradient. The pathways represented have been selected among the top five pathways for each stimulation due to their potential links with inflammation and viral control. **(A)** DRD1-induced Th1 and Th2 cell differentiation, **(B)** PI3K-AKT signaling pathway, and **(C)** DRD1-induced antigen processing and presentation pathways.

**Figure 12:**
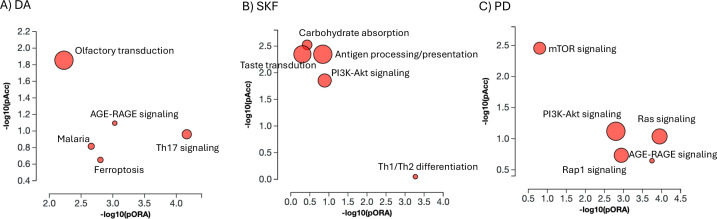
Pathway perturbation vs. overrepresentation. The top 5 pathways for **(A)** DA, **(B)** SKF38393, and **(C)** PD168077 are plotted in terms of overrepresentation on the *x*-axis (pORA) and the total pathway accumulation on the *y*-axis (pAcc). Each pathway is represented by a single dot, with significant pathways shown in red, and the size of each dot is proportional to the size of the pathway it represents. Both *p*-values are shown in terms of their negative log (base 10) values.

**Figures 9**, **10** and **11** show the schematic organization of the perturbed pathways by stimulus with color-coded genes found in transcriptional patterns. DA increased Th17 signaling pathway (*p* = 0.004), with a contribution of pro-inflammatory genes S100A8 and S100A9, which activate HIV-infected cells via RAGE (2), as well as AP-1 as a transcription factor with ties to IL6, CCL2, and CXCL8 (**Figure 9**). AGE-RAGE signaling was the second most significant pathway triggered by DA (**Figure 12**).

The comparison of signatures and overlapping pathways has suggested that, despite the fact that S100A8 and A9 were not significantly upregulated by PD168077 (**Figure 10**), the AGE-RAGE signaling was also significantly perturbed via DRD4 (*p* = 0.021) (**Figure 10A**), in agreement with previous findings in DA. In addition, the Ras signaling pathway was strongly affected by DRD4 (*p* = 0.004), with potential consequences to gene transcription (**Figure 10B**) and inflammation. In these figures, it is important to appreciate the location and directionality of the genes exhibiting change within each pathway as a way to estimate the importance of those changes to the biological process.

SKF, on the other hand, affected pathways in inflammation, and immune responses, such as Th1/Th2 differentiation (*p* = 0.005) (**Figure 11A**), antigen processing and presentation (*p* = 0.028) (**Figure 11B**), and PI3K-Akt signaling pathways (*p* = 0.044) (**Figure 11C**), where PP2A shows downregulation with upregulation of Akt, with additional increase of genes involved in cell survival. This pathway is downstream the activation of GPCRs (42), such as DRDs.

### 3.7 Upstream regulators

**Figure 13:**
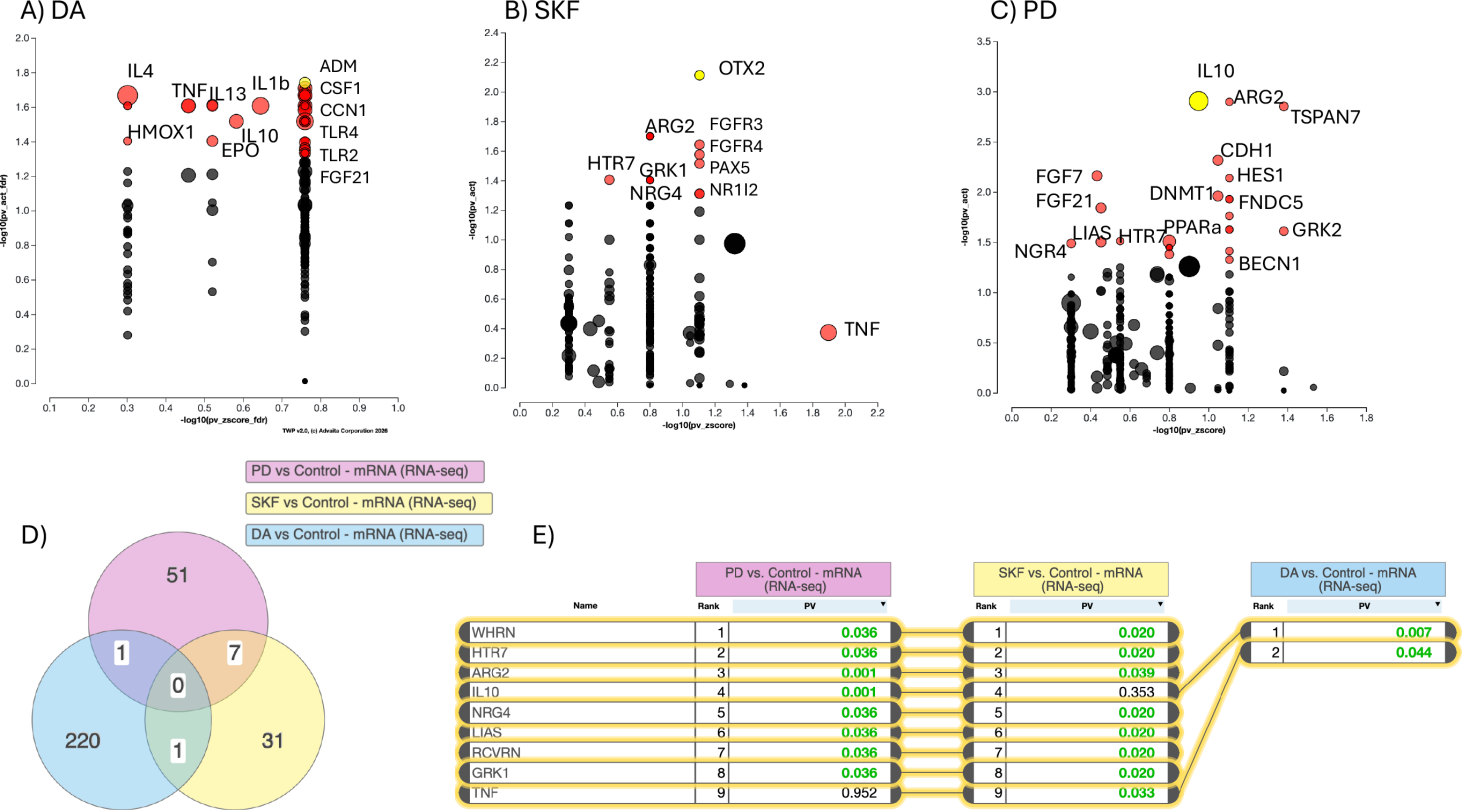
Predicted upstream activated regulators. Under the hypothesis of activated upstream regulators, the number of consistent differentially expressed (DE) genes downstream was compared to the number of measured target genes, using an overrepresentation approach in iPathwayGuide. **(A)** Predicted regulators in DA stimulation. **(B)** Predicted regulators in SKF38393—SKF stimulation. **(C)** Predicted regulators in PD168077—PD stimulation. Dots represent upstream regulators. The *x*-axis position is the log of the FDR-adjusted *p*-value based on the *z*-score. The *y*-axis position is the log of the overrepresentation *p*-value based on the number of consistent DE targets. The size of each dot represents the number of consistent DE genes for each regulator. The yellow color dots signal the top predicted regulators for each stimulating condition. The red color dots represent the regulator above the significance cutoff. **(D)** Venn’s diagram showing the number of predicted and overlapping regulators by condition. **(E)** Rank diagram showing overlapping regulators between any two conditions, where significant *p*-values are in green.

Despite the limited overlap in signatures and pathways between DA and selective DRD stimulations resulting in latency reversal, we tested whether the prediction of activated upstream regulators could result in common components. **Figure 13** shows these predictions based on the enrichment of differentially expressed (DE) genes and the network of regulatory interactions formed between them using iPathwayGuide (Advaita), ranked by *p*-value and number of targets. The top predicted regulator in DA-stimulated cells was adrenomedullin (ADM). Other upstream regulators with pro-inflammatory potential were identified in DA-stimulation conditions, such as CSF1, CCN1, TLR2, TLR4, IL1b, and HMOX1, but also regulatory cytokines such as IL4, IL13, and IL10 (**Figure 13A**). The selective stimulation via DRD1 had the orthodenticle homeobox 2 (OTX2) as a top predicted regulator. Other hits included molecules with immune-regulatory functions such as TNF, the fibroblast growth factor receptors (FGFR) 3 and 4, PAX5, ARG2, NR1I2 and NRG4, GRK1, and HTR7 (**Figure 13B**), some of which shared with DRD4 (**Figure 13E**). IL10 was the top regulator of gene expression phenotypes induced via DRD4, along with ARG2, GRK2, ARG2, TSPAN7, CDH1, HES1, FNDC5, DNMT1, PPARA, HTR7, LIAS, FGF7, FGF21, and NGR4. It is interesting that DA shared one regulator with either DRD1- or DRD4-selective stimulations, but the two receptors had seven upstream common regulators (**Figure 13D**). In addition, there were no common predicted regulators satisfying all three conditions, corroborating findings of minimal overlap. The prediction summarized in **Figure 13E** indicated that DA may share IL10 as a regulator with DRD4-selective activation by PD198077, and TNF as a regulator with DRD1-selective activation by SKF38393. IL10 was a top regulator in DRD4 stimulation.

### 3.8 Characteristics of latency reversal by cluster

**Figure 14:**
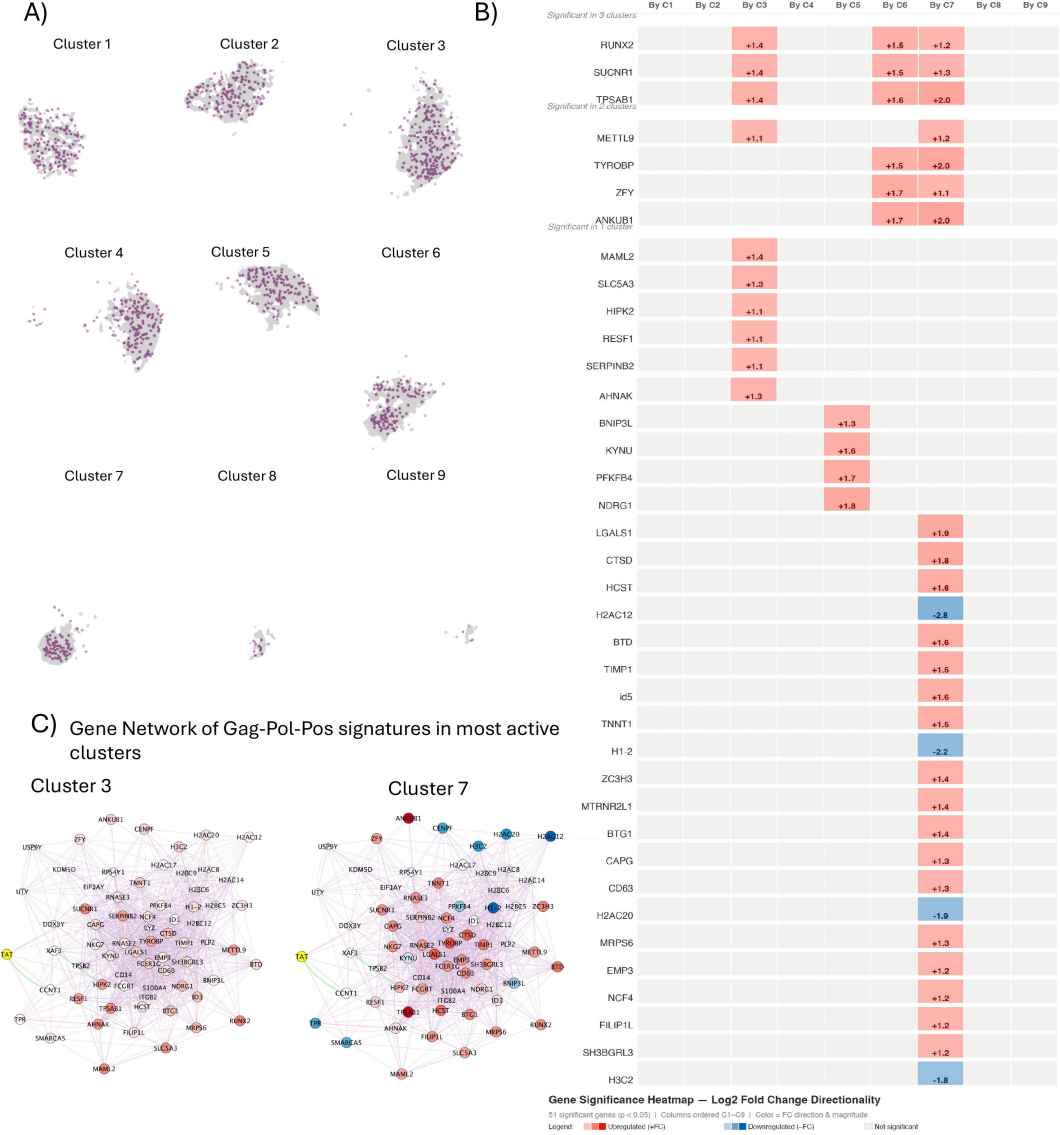
Characterization of active HIV-transcribing gene subsets by cluster. **(A)** Visualization of HIV Tat-Gag-pol positively transcribing cells gated by Lasso in Loupe software (gray). Dots indicate cells with positive transcription of DRD1 and DRD4. **(B)** Heatmap with fold change (FC) directionality, color-coded with red indicating upregulated (positive Log2 FC) and blue indicating downregulated (negative Log2 FC) and darker shades indicating higher magnitude. The FC value is shown inside each significant cell. **(C)** Gene network analysis of signatures in cells actively transcribing HIV genes in the two most active clusters (clusters 3 and 7).

While cells with positive HIV gene transcription was enhanced in all clusters following DA or selective stimulation, one remaining question was whether cells experiencing latency reversal looked alike in the different clusters. To address this question, a feature filter was introduced to identify Tat-Gag-pol-positive transcription followed by a LASSO strategy and reanalysis of DE of Pos vs. Neg cells by cluster, normalized to the whole average dataset (**Figure 14A**). The approach generated a visualization of only actively transcribing Gag-pol-positive cells by cluster, and overlapping dots indicate cells with significant expression of DA receptor (DRD1–DRD5) genes. The visualization suggests that cells transcribing DRDs co-localize with the populations with stronger viral gene transcription (**Figure 14A**), further supporting observations. The analysis indicated that there were no overlapping significant signatures in cells experiencing latency reversal across clusters. However, in the three clusters with higher overall transcriptional activity (clusters 3, 6, and 7), 3 common genes were significantly upregulated in cells actively transcribing HIV genes (**Figure 14B**). These genes were runt-related transcription factor 2 (RUNX2), succinate receptor 1 (SUCNR1), and tryptase alpha/beta 1 (TPSAB1). METTL9, TYROBP, ZFY, and ANKUB1 were upregulated in two of the same clusters. Cluster 7, stimulated by DA alone, was the most active cluster, followed by cluster 3 (**Figure 14B**). Gene network analysis of the genes in Tat-Gag-pol-positive cells in these two most active clusters suggested interactions with histone linkers and chromatin structure controllers such as H2AC17, H2AC20, H1–5, and others, which remained low or were further downregulated (**Figure 14C**).

**Figure 15:**
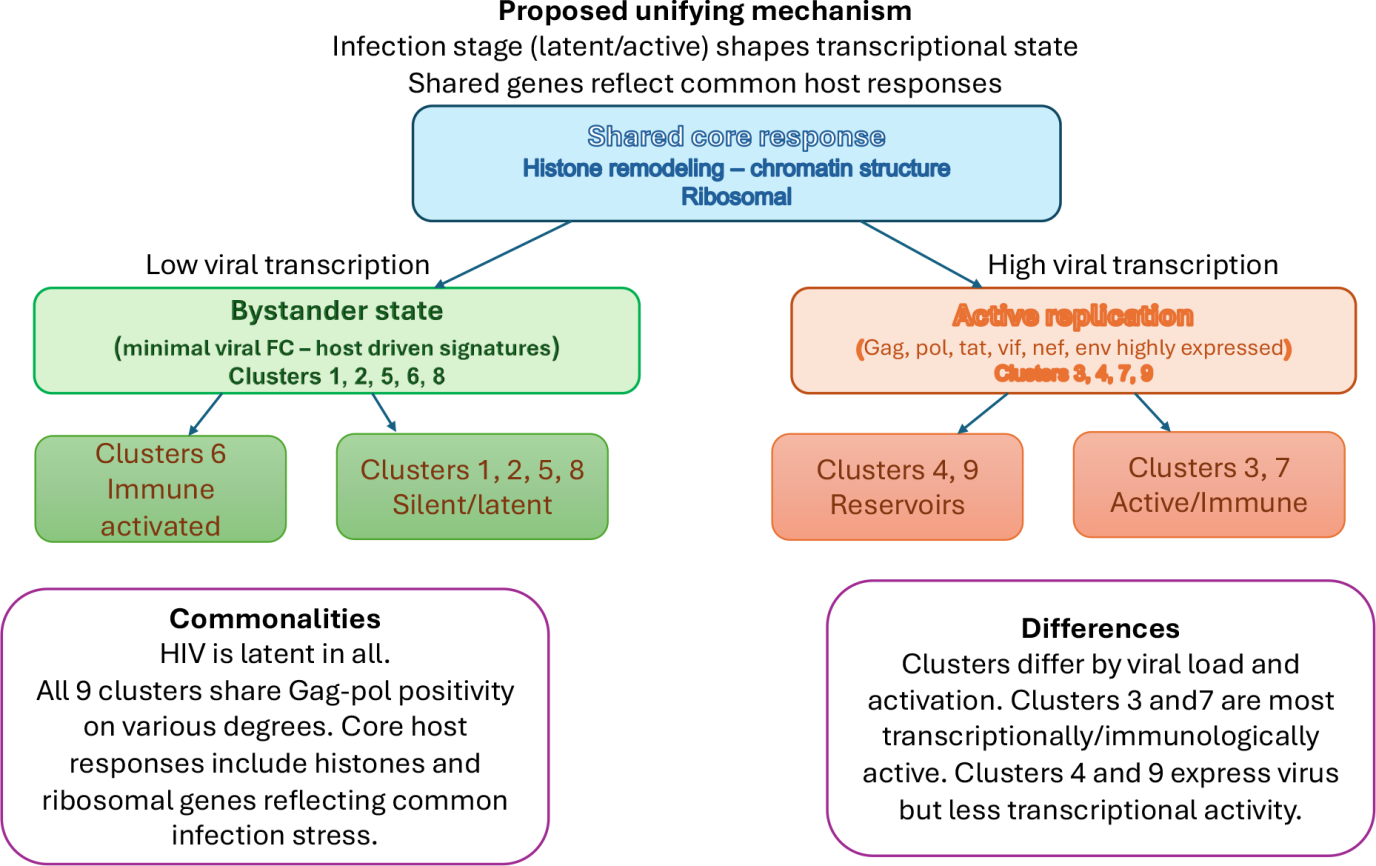
Summary of characteristics of cells with positive HIV gene transcription by cluster, with commonalities and differences. Gag-pol-tat-positive cells were extracted from each of the nine clusters following LASSO feature selection, and arranged according to their proposed stage in the HIV infection (latent/active), inferred from viral gene fold changes and the number and identity of significantly differentially expressed host genes (*p* < 0.05). The shared core response (blue, top) represents transcriptional programs common across multiple clusters, including histone remodeling, chromatin structure components, and ribosomal genes, reflecting conserved host responses. Clusters are further stratified into two main branches based on viral activity: low and high transcription characterized by fold changes in HIV structural and regulatory genes (gag-pol, env, tat, and vif—not p24; bystander clusters were characterized by predominantly host-driven transcriptional signatures). Silent clusters show no statistically significant HIV gene expression changes and are proposed to represent latently infected cells. Commonalities and differences between HIV+ transcribing cells by cluster are highlighted in clear boxes.

A summary of the commonalities and differences between Gag-pol-Tat-positive cells by cluster can be found in **Figure 15**.

## 4 Discussion

This study was designed to help understand the contribution of signaling downstream of DA receptors to HIV latency reversal in conditions of exposure to high levels of the neurotransmitter DA, such as in the brain of stimulant substances like Meth. We focused on the selective activation of DRD1 and DRD4, both of which can increase transcription of HIV genes in a cell line model of innate immune latency, as previously described (1, 2). Using a single-cell approach to identify subpopulations that are responsive to stimuli with transcription of viral genes, we compared expression patterns between DA and selective agonists to DRD1 and DRD4, to determine whether the role of receptors in DA-induced pathways and learn more about viral control. These receptors were selected because they are most abundantly expressed by U1 cells and because of the effects of selective agonists on p24, and transcription, which replicated our previous reports using bulk RNAseq (1, 2). Thus, the results in this study confirm previous observations regarding the importance of certain DRDs to the myeloid response in the context of a hyperdopaminergic environment, but it differs from our previous approaches, because the single-cell-based transcriptome method allowed the distinction and digital sorting of the specific cellular events that have detectable HIV gene transcription, as opposed to the cells in which the integrated virus remains silent.

We found that even though the increase in p24 was achieved by DA, both via DRD1 and DRD4, the cluster dynamics, signatures, and pathways were somewhat different, suggesting that HIV gene transcription can be turned on for different reasons in the context of high levels of DA, depending on receptor expression, signaling characteristics, and availability. Importantly, DA triggered unique profiles represented by cluster 7, with strongly significant gene expression differences including in HIV gag-pol. While the transcriptional and functional gene network profiles in cluster 7 may result from combinatorial receptor signaling, immune-related annotations suggested a significant contribution of this cluster to the observed DA-induced inflammatory sets of genes in U1 latent cells and potentially other outcomes relevant for HIV in the brain (7). Yet, selective stimulations showed distinct and overlapping aspects of pro-inflammatory actions.

Interestingly, despite converging pro-inflammatory activity in HIV gene-positive clusters, only 5 genes were common between DA, DRD1, and DRD4 upregulation, which rendered no common predicted upstream regulators. Added to the fact that DA modified less genes than the individual selective stimuli, the result suggests that the DA receptors cross-regulate when simultaneously activated on myeloid cells, with regulatory elements that are not present when one single receptor is activated. DRDs are G-PCRs, and the different subtypes, e.g., Type I (DRD1 and DRD5) and Type II (DRD2, DRD3, and DRD4), often work in opposition to manage cyclic adenosyl monophosphate (cAMP) (43). Thus, phenotypes may also result from a complex interplay between receptor availability and affinity.

We previously described that a pro-inflammatory mediator, MRP8/14, which is induced by innate immune cells stimulated with DA, can replicate the effects of DA on reversing latency in U1 cells through its binding to RAGE (2, 7). However, despite the fact that both DRD1- and especially DRD4-selective agonists increased HIV gene transcription, the effect of single receptors did not correlate with a significant increase in the transcription of the MRP8/14 genes S100A8 and S100A9. Yet, HIV transcription in PD198077-stimulated cells mapped to clusters was also identified in DA stimulation (clusters 3, 5 and 6) and to the AGE-RAGE pathway, suggesting strong similarities with the native neurotransmitter and the signaling elicited by MRP8/14 via RAGE (2). In cells stimulated via DRD1, HIV gene-positive cells were more frequent in clusters 2 and 4, which were not represented in DA stimulation. This indicates that latency can be reversed by DA receptors, but the different receptors or combinations of receptor signaling may do it so for different but converging reasons, where MRP8/14 is one of the contributing factors. However, other factors activating advanced glycation end (AGE) products via their RAGE receptors may also be a trigger. Others have reported that S100A8 serves as an inflammatory alarmin that can also target TLR4, causing cellular metabolism to shift toward glycolysis, which leads to the reactivation of replication-competent viral particles in macrophages (44), in a cross-talk with RAGE pathway (45). Pharmacological blockage of TLR4 was able to prevent MRP8/14-induced latency reversal, when the antagonist TAK242 was added, although only at high doses (2). The results suggest that signaling via DRD4 may be a main contributor in turning HIV gene replication on by the RAGE pathway, potentially activated via TLR4.

It is overall interesting that there are very defined transcriptional actions that suppress or activate sets of genes in the same cells. One example of this is the downregulation of the gene encoding the enzyme PP2A, along with an increase in Akt, within the PI3K-Akt pathway, downstream GPCR activation (42). PP2A appeared as an isolated signature of SKF38393, highlighted by pathway enrichment strategies but not connected in gene network analyses. Suppression of PP2A by HIV Vif has been linked to cell cycle arrest in CD4 cells (46). The decrease in PP2A can be a host–virus factor contributing to latency reversal, since it has been shown that its recruitment downstream type I IFN signaling is critical for containing viral transcription and release (47, 48).

DRD receptors are G-PCRs. D1-like receptors (DRD1 and DRD5) most likely couple to G proteins of the Gs subfamily that stimulate the adenylyl cyclases and cAMP production, while the D2-like receptors (DRD2, DRD3, and DRD4) couple to members of the inhibitory Gi family members (Gi/Go) (49). Gs engagement classically activates adenylyl cyclase and increases cAMP, which has been shown to reverse latency in this same model (50). Interestingly, DRD1 stimulation induced phenotypes and pathways that differed from PD168077and from DA, with more perturbations characterized by immune and inflammatory pathways. Thus, the stronger overlap between DA and PD168077 may indicate that DRD4 is the predominant GPCR signaling in latent myeloid cells in this model, in regard to latency reversal.

CD36 was a potential interesting marker because it is a fatty acid transporter with a role in neuroinflammation (51) that appeared consistently in correlation with positive HIV gene transcription in cluster-specific gene network analyses. However, we did not confirm its role in activating HIV, and effects on HIV have not been described. In fact, SSO, the inhibitor used in our study alone, increased p24, despite having been described to inhibit inflammation in microglia (51). SSO influences the mitochondrial respiratory chain (52) by causing a suppression that could potentially mimic the profile of cluster 5, with low viral transcription while mitochondrial genes are downregulated. Nevertheless, the ability of SSO to induce a potential regulatory immune phenotype (53) was not confirmed in U1 promonocytes exhibiting latent HIV, indicating that CD36 is not a good marker to monitor or explain the phenomenon of reversal by DA. Yet, CD36 has been investigated as an alternative receptor for HIV entry and a co-factor in the release of virus in macrophages (54), suggesting that its upregulation may influence other aspects of the infection in myeloid cells.

The prediction of regulators provided insights. A major DA-induced regulator was ADM, which plays a role in anti-inflammatory, vascular, and antimicrobial functions, and appears among signatures of protection against dementia (55, 56). The DRD1 predicted top regulator OXT2 is a transcription factor that not only plays a role in the brain, influencing the proliferation and differentiation of dopaminergic neuronal progenitor cells during mitosis, but also plays a critical role in controlling chromatin structure (57–59). OXT2 is critical in brain development and is not usually expressed by myeloid cells. It is also interesting that despite perturbing pro-inflammatory pathways and triggering HIV viral transcription, the overall stronger regulator identified via DRD4 was IL10, which was shared with DA, suggesting that this receptor may contribute to the immune suppression effects of DA, and that latency reversal is regulated by a different set of rules in smaller subsets of cells. The cluster analysis resolves this issue, first by showing that DA and its receptors reverse latency not in all but just a fraction of cells, then by indicating the role of different receptors in functional diversity, mimicking and potentially explaining the heterogeneity of populations that occur *in vivo*. Finally, given that major clusters drive overall phenotypes, minoritarian clusters may be masked, even if enriched and bearing important biological contributions. For instance, clusters 1 and 2 contain the largest number of cells and phenotypes of gene downregulation that make them fundamentally different from cluster 3, and especially cluster 7, which show the strongest gene upregulation and inflammatory phenotypes, driving HIV gene transcription by higher transcriptional activity. The relative abundance of clusters with distinctive gene expression profiles and properties in a cell type-, receptor subtype-, microenvironment-, and stimulus-dependent manner can modify responses drastically and drive interpretations of overall outcomes, such as in studies where DA is an anti-inflammatory and immune suppressor (60). Phenotypes of activation or suppression following exposure to DA are therefore largely context-dependent. Understanding the conditions is critical for HIV latency and for harnessing this knowledge to eradicate the virus.

The unifying principle is that all nine clusters derive from latently infected cells, and all have representation of positive HIV gene transcription to various fold change degrees and transcriptional signatures that may reflect where each cell population stands along that spectrum. One commonality is that every HIV gene-positive cell, regardless of cluster, expresses a core host response involving histone remodeling, chromatin structure, and ribosomal changes. For instance, RUNX2, SUCNR1, and TPSAB1 appear across clusters 3, 6, and 7, suggesting a common pathway and innate inflammatory signatures of shared infection stress. Cluster 7 stands out due to the broadest host response (43 significant genes), followed by the cluster 3 shared between DA- and DRD4-selective stimulation. These two transcriptionally active clusters were most significantly activated by DA, further supporting the role of this neurotransmitter in aggravating viral infection and inflammation, explaining previous observations in the brain of drug users (13). Clusters 6 and 7 show overlap of signatures, indicating a common core in host response genes, despite differing in other ways, including fold change of HIV viral genes. These characteristics suggest that cell populations that are actively transcribing virus at different levels can mount a similarly strong immune counterresponse. On the other hand, as previously mentioned, the activation by DA via a combination of receptors that is predominant in cluster 7 may trigger a broad response that stochastically includes transcription of randomly integrated HIV gene sequences. Cluster 9 shows strong HIV gene transcription, despite low p24 and low transcriptional activity, suggesting that this cluster is a candidate for a potential reservoir phenotype. Clusters 1, 2, 4, and 8 may represent cells that remain, for the most part, latent, or where viral and host transcriptional programs are suppressed. A schematic representation of these principles can be found in **Figure 15**.

The limitations of a system using cell lines include the possibility that different clusters may represent different phases in the activation process, owing to timing, replication status, or kinetic differences not addressed here. Still, differences and similarities are useful to understand the processes associated with active transcription, and to gain insights into regulatory mechanisms that can be targeted to eliminate the latent reservoir. Moreover, despite the limitations, our studies inform future experiments using myeloid cells isolated from infected subjects, which consist of rare events, and for which knowledge of the correct signatures can be helpful to identify the reservoir.

As HIV disrupts the DA system in the brain (61–64), DA-driven behaviors, especially stimulant use, can increase HIV risk and worsen HIV neurological outcomes (14). Observations from human cohorts have been confirmed in experimental models (13, 15, 65), indicating that effects of high DA levels lead to higher viral replication and spread. The effects on latency have also been described in this model (1). The experiments discussed here indicate a role for DRD4 and DRD1 via different pro-inflammatory pathways. The results provide important insights for the development of drugs that interfere with DA signaling to control HIV latency and replication with therapeutic goals, particularly in populations of substance users.

While Meth was a major contextual motivation for this work, the implications of these findings extend beyond stimulant use disorders. Elevation of mesolimbic DA is a shared neurobiological feature of multiple substance use disorders, including cocaine, opioid, and alcohol, albeit through distinct upstream mechanisms. Cocaine increases extracellular DA by blocking the transporter (66), opioids disinhibit dopaminergic neurons in the ventral tegmental area via μ-opioid receptor-mediated inhibition of GABA interneurons (67), and alcohol enhances DA release in reward circuitry (68). Thus, dopaminergic receptor engagement in brain-resident myeloid cells may represent a convergent downstream pathway across diverse substance use disorders.

In the hyperdopaminergic environment triggered by stimulant drugs, immune cells, particularly cells that are HIV targets, are not just bystanders. Drugs such as Meth have effects that can be direct, or mediated by reactive oxygen species, cAMP, or mitochondrial dysfunction (7, 69–71). Interestingly, Meth and DA play distinct roles on triggering the expression of genes that are relevant to HIV infection, for instance, CCR5 (7), with implications to viral entry and spread. The multiple ways HIV targets can be affected in the context of substance use introduce molecular confounders, but these can be sorted by an *in vitro* system such as the one used here, with a specific focus on latency. The observation that cells with active HIV transcription cluster differently when exposed to DA and via its receptors is an important evidence of the power of neurotransmitters such as DA to influence non-neuronal cells, HIV targets, and infection in the brain of drug users.

In parallel, accumulating evidence indicates that HIV transcription and protein expression can persist despite suppressive ARV therapy, even in the absence of detectable plasma viremia (72). In the CNS, microglia and macrophages are recognized as persistent reservoirs despite durable therapy (4). In this context, our observation that DRD1 and DRD4 activation promotes HIV gene transcription in a subset of latent myeloid cells suggests that recurrent dopaminergic surges—regardless of the precipitating substance—could modulate HIV transcriptional activity within CNS reservoirs.

Importantly, we observed baseline heterogeneity in HIV transcription even in unstimulated cells, with DA and selective receptor agonists increasing the frequency and magnitude of transcriptionally active states rather than converting a uniformly silent population. This state-dependent modulation aligns with emerging models of latency as a dynamic equilibrium rather than an all-or-none phenomenon. In individuals with substance use disorders characterized by repeated dopaminergic activation, episodic enhancement of HIV transcription in microglia or macrophages could contribute to sustained neuroinflammation and may partially explain worse neurocognitive outcomes observed across stimulant and other substance use disorders in people with HIV (17, 19).

Overall, the results suggest that although DRD1- and DRD4-mediated signaling can promote pathways that affect inflammation and ultimately lead to latency reversal in subsets of U1 cells, replicating the effects of DA, the pathways and upstream regulators associated with such effects may differ between stimuli. The lack of overlap in phenotypes between DA and the selective receptor stimulations is somewhat surprising. Yet, components of the AGE-RAGE pathways overlapped particularly between DA- and DRD4-mediated stimulation, and with elements of this pathway represented in cluster profiles linked to increased viral gene transcription. DRD1-mediated signaling increased HIV gene transcription and activated immune inflammatory pathways. The results suggest that DA triggers phenotypes that differ from the stimulation via individual receptors, but may result from a combinatorial effect converging on the neuro-dopaminergic regulation of pro-inflammatory responses.

Interestingly, the transcriptional profiles of cells with positive HIV gene transcription between clusters indicated a partial overlap linked to stronger transcriptional activity, suggesting that latency reversal results from changes in chromatin structure due to cellular activation. The random character of the viral integration process that precedes latency may contribute to the diversity in phenotypes upon reversal. Yet, most consistent features include RUNX2, a regulator of myeloid cell differentiation (73) triggered via DRD1 (74), and SUCNR1, a regulator of inflammatory phenotype that is sensitive to metabolic states (75, 76). TPSAB1, a gene generally present is mast cells (77), was another signature present in HIV transcribing cells in the three most active clusters, without any known role in HIV, latency, or DA stimulation.

Targeting DA receptor pathways represents a genuinely promising and underexplored therapeutic frontier, particularly at the intersection of HIV, neuroinflammation, and substance use. DA receptors are druggable targets. For instance, DRD2 antagonists are currently explored in psychiatric and neurological conditions (78–80). In our model, DRD2-targeting compounds do not affect viral outcomes (7). However, given that immune cells express other functional DA receptors that modulate viral replication, immune activation, and latency reversal, the DA–immune axis represents a potential disease-modifying pathway where interventions could alter the underlying biology of HIV persistence and opportunities for repurposing. More studies in human and animal models are needed to explore this possibility.

Taken together, our results support a broader framework in which dopaminergic receptor signaling serves as a mechanistic bridge between substance use disorders and modulation of HIV reservoir activity in the CNS, as shown in this study, via the activation of latent cells exhibiting integrated virus leading to active HIV gene transcription. Rather than being Meth-specific, the pathway-level biology described here may reflect a convergent neuroimmune interface linking several levels of reward circuitry activation to HIV transcriptional dynamics in brain myeloid cells and highlights DA receptor pathways as potential targets for therapeutic modulation.

## Data Availability

The datasets presented in this study can be found in online repositories. The names of the repository/repositories and accession number(s) can be found in the article/**Supplementary Material**.

## Supplementary material

Supplementary Figure 1 P24 Levels - P24 was measured by ELISA in U1 latent cells **(A)** at baseline and in the presence of the latency reversal agent PEP005 (1 uM). **(B)** Effect of the CD36 antagonist Sulfosuccinimidyl oleate (SSO) for 12 hrs, alone or in the presence of DA at the indicated concentrations.
