# Supplementary figures and images for: Distinct roles of dopamine receptors in HIV latency reversal in a myeloid cell model
Source: Front Immunol. 2026 May 28;17:1817754. doi: 10.3389/fimmu.2026.1817754 (PMC13253437; doi:10.3389/fimmu.2026.1817754)

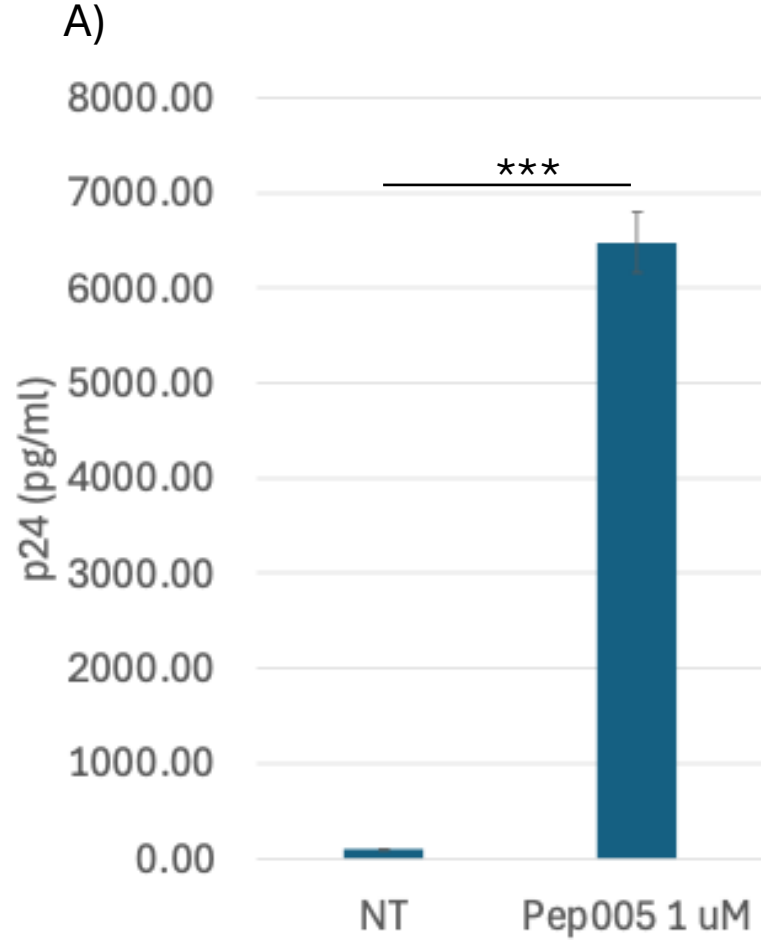

\*p<0.05  
\*\*p<0.01  
\*\*\*p<0.001

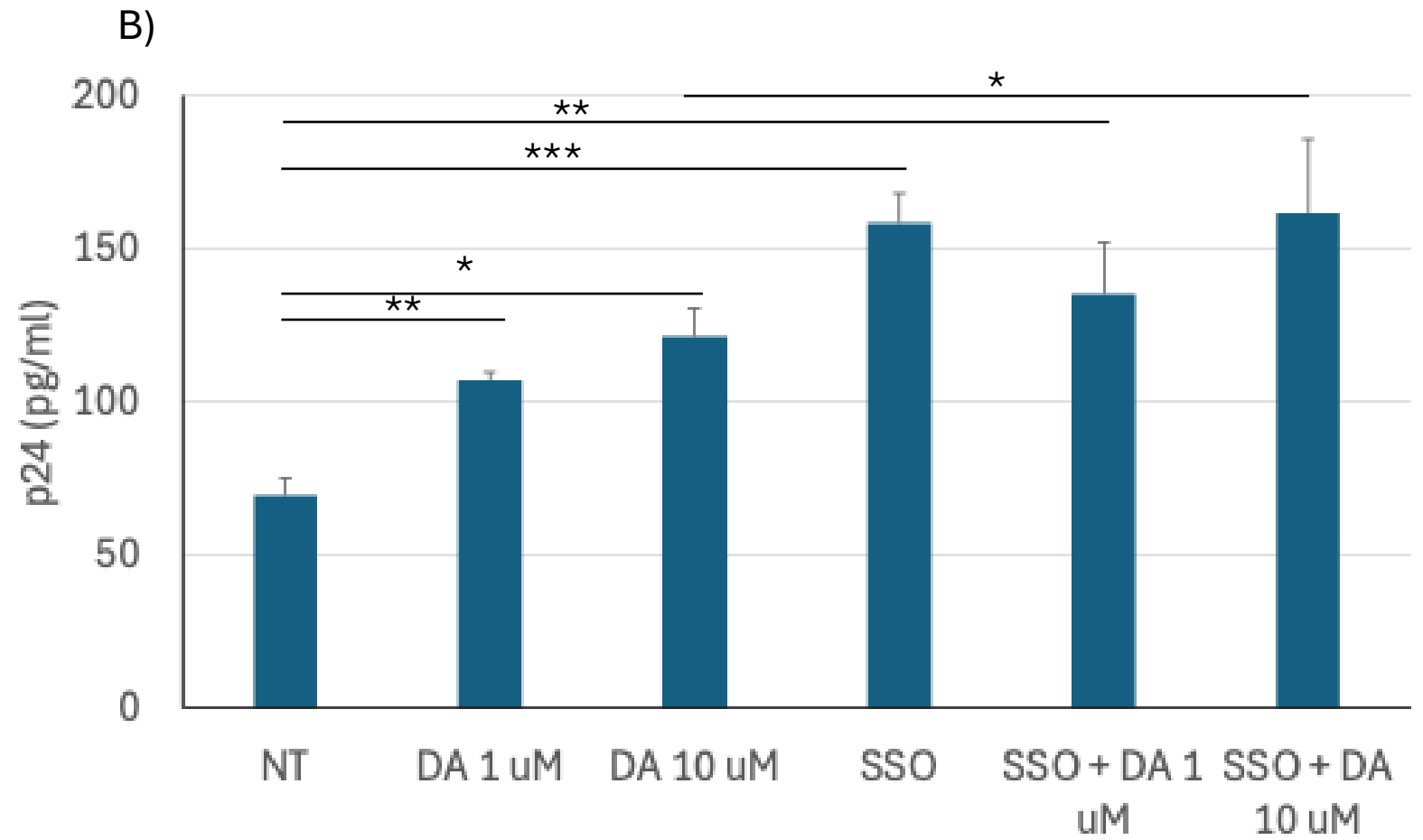

Supplement: Supplementary Figure 1 — P24 Levels - P24 was measured by ELISA in U1 latent cells (A) at baseline and in the presence of the latency reversal agent PEP005 (1 uM). (B) Effect of the CD36 antagonist Sulfosuccinimidyl oleate (SSO) for 12 hrs, alone or in the presence of DA at the indicated concentrations. [file SupplementaryFile1.pdf]
